# Squares of different sizes: effect of geographical projection on model parameter estimates in species distribution modeling

**DOI:** 10.1002/ece3.1838

**Published:** 2015-12-17

**Authors:** Lara Budic, Gregor Didenko, Carsten F. Dormann

**Affiliations:** ^1^ Biometry and Environmental System AnalysisUniversity of FreiburgFreiburgGermany

**Keywords:** climate niche model, equal area projection, GLM, long‐lat projection, equal‐area projection, species distribution model

## Abstract

In species distribution analyses, environmental predictors and distribution data for large spatial extents are often available in long‐lat format, such as degree raster grids. Long‐lat projections suffer from unequal cell sizes, as a degree of longitude decreases in length from approximately 110 km at the equator to 0 km at the poles. Here we investigate whether long‐lat and equal‐area projections yield similar model parameter estimates, or result in a consistent bias. We analyzed the environmental effects on the distribution of 12 ungulate species with a northern distribution, as models for these species should display the strongest effect of projectional distortion. Additionally we choose four species with entirely continental distributions to investigate the effect of incomplete cell coverage at the coast. We expected that including model weights proportional to the actual cell area should compensate for the observed bias in model coefficients, and similarly that using land coverage of a cell should decrease bias in species with coastal distribution. As anticipated, model coefficients were different between long‐lat and equal‐area projections. Having progressively smaller and a higher number of cells with increasing latitude influenced the importance of parameters in models, increased the sample size for the northernmost parts of species ranges, and reduced the subcell variability of those areas. However, this bias could be largely removed by weighting long‐lat cells by the area they cover, and marginally by correcting for land coverage. Overall we found little effect of using long‐lat rather than equal‐area projections in our analysis. The fitted relationship between environmental parameters and occurrence probability differed only very little between the two projection types. We still recommend using equal‐area projections to avoid possible bias. More importantly, our results suggest that the cell area and the proportion of a cell covered by land should be used as a weight when analyzing distribution of terrestrial species.

## Introduction

**Figure 1:**
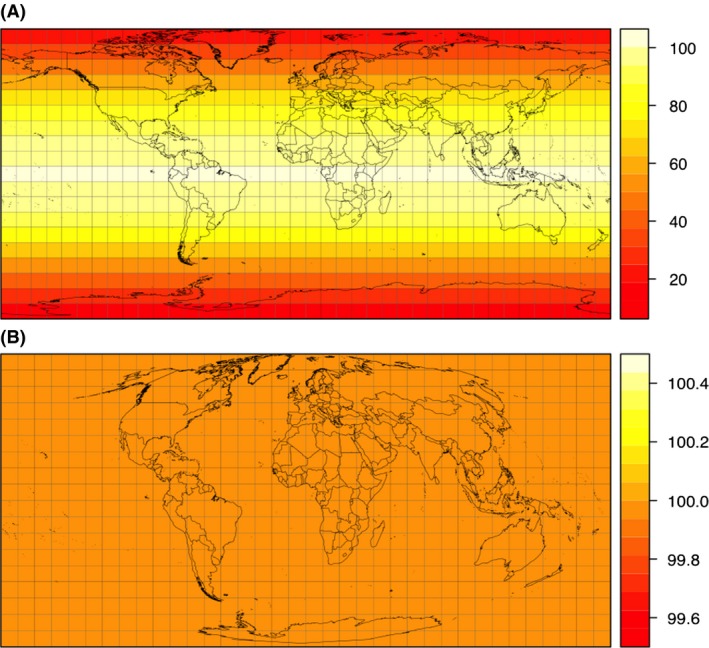
The two projections compared in this study. (A) long‐lat projection, also called *plate carée* projection. (B) equal‐area Mollweide projection. Dark red means smaller cell area. White cells in (A) are approximately same size as cells in (B). Note that polar regions harbor many more cells in long‐lat compared to Mollweide projection. Grid is 9${}^{\circ }$ × 9${}^{\circ }$ and for illustration only.

Geographers have devised many different geographical projections to address the challenge of flattening the surface of our 3‐dimensional world onto 2‐dimensional maps. When representing data at continental to global scale, the main decision is whether to present a map with straight, parallel meridians and circles of latitude, intersecting each other perpendicularly (e.g., Mercator projection), or with curved lines. The former, “long‐lat” projections yield world maps appealing to the human eye for their plane appearance; however, the areas they depict are distorted, and more so toward the poles (Mulcahy and Clarke 2001). The extreme opposite uses equal‐area projections, for which every cm${}^{2}$ on the map covers the same area on the globe, but they do so at the expense of distorting circles of latitude and longitude (Fig. 1). Compromise projections try to strike a balance between both extremes. Mathematically, each projection can be transformed into each other, and the geographical coordinates associated, for example, with species locations can hence be displayed on any projection.

**Figure 2:**
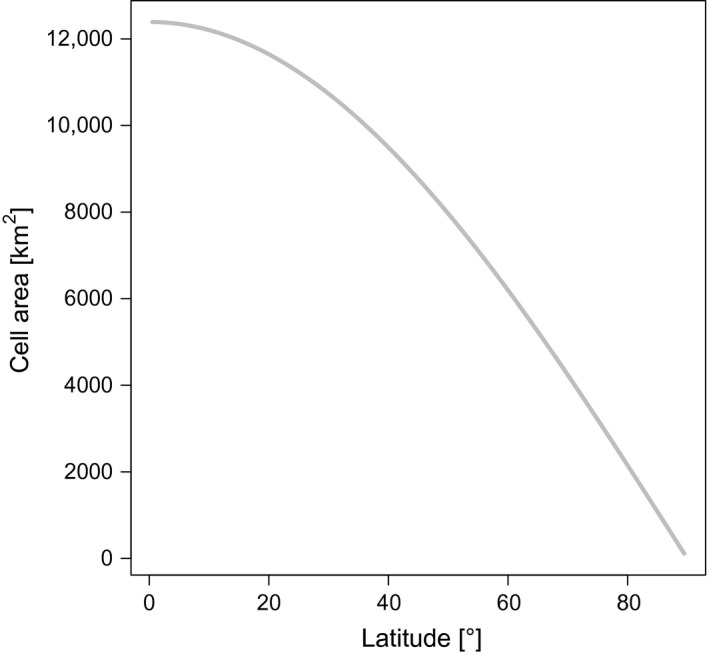
Area of a 1${}^{\circ }$ × 1${}^{\circ }$ cell in equirectangular projection from equator to the pole.

There is, however, a potential effect of geographical projection when rasterizing environmental data to one or the other projection and using these data for spatial statistical analyses. Many global datasets, such as worldclim (Hijmans et al. 2005) or Global Circulation Models, use a long‐lat raster: 5${}^{\circ }$, 1${}^{\circ }$, 1/2${}^{\circ }$, 10' or alike. That means that a 1${}^{\circ }$ × 1${}^{\circ }$ cell at the equator has an area of approx. 110 × 110 km, while toward higher latitudes the same 1${}^{\circ }$ × 1${}^{\circ }$ cell shrinks to effectively 0 at the north and south pole (Fig. 2).

The question we address in this study is whether such change in cell size matters for the analysis of species distributions. For example, for studying long‐distance bird migration routes, distance and direction might be the most important parameters (Gudmundsson and Alerstam 1998), while for species turnover analysis, an equal‐area representation might be more important (Gaston et al. 2007). We would argue that in species distribution or niche modeling (Peterson et al. 2011), preserving the correct area size occupied by the species is more relevant than preserving accurate shapes or angular relationships. The reason is that in the statistical analysis, each raster cell represents one data point. If the area of raster cells is different, they should be given different weight in the analysis to allow an equal contribution of all segments of a species' range to the distribution model. An increase in the number of cells with latitude in long‐lat projection translates into an increase of sample size and, at the same time, a decrease of range area represented in each sample. Not weighting for the area leads to a disproportionate contribution of northernmost conditions to the model. All else being equal, cell area also correlates with both environmental heterogeneity and occurrence probability of the target species (see Keil and Hawkins 2009, for in depth discussion).

Today, most large‐scale species distribution studies work with equal‐area raster data, but a large number of studies used (and still uses) degree‐based raster data (an incomplete sample spanning the last 15 years: Cumming and Road 2000; Lovett et al. 2000; van Rensburg et al. 2002; Bonn et al. 2004; Hartley et al. 2006; Holmgren and Poorter 2007; Kriticos and Leriche 2010; Veloz et al. 2012; Bled et al. 2013; Botts et al. 2013; Gwitira et al. 2013). It is unclear how much the difference in projections influences species distribution modeling, specifically the estimates of model parameters, the shape of the functional relationship between environmental predictor and occurrence probability, and hence, the prediction made with such models, either to future climates or other regions.

To test the effect of long‐lat versus equal‐area projection, we compare analyses of the same data at two different projections (the original long‐lat projection, and equal area: Mollweide). As test cases we analyzed IUCN range data of 12 Northern Hemisphere ungulates, because at higher latitudes the distortion is largest and we can therefore use our findings as a worst‐case situation. In addition to the two projections, we also investigate the use of cell area as regression weight to compensate different cell sizes in the long‐lat projection. During a pilot study, we found that such weighting can have substantial effects, and we decided to also, as a third factor, include a weighting for the actual area covered by land in each cell, thereby down‐weighting coastal cells in both projections. This yields six different models (see Table 2).

## Methods

### Species data

The study area was the holarctic biogeographical realm as defined by UNEP‐WCMC 2004. We selected species occurring at different latitudes to investigate whether projectional distortion may be primarily affecting models of northern species. The 12 holarctic ungulates cover a large span of range sizes, with some having a coastal and others a purely continental distribution (Table 1).

**Table 1:** Ungulate species used in this study, sorted by range size ($10^{6}$ km${}^{2}$). Ranges without coastal distribution are marked with an asterisk. Original range sizes represent IUCN data extracted to L (long‐lat) and M (Mollweide) projections. Slight discrepancies are caused by thresholding species presence to more than 10% of cell's area. Predicted range sizes are based on predicted occurrence probabilities thresholded at prevalence level multiplied by the cell's area in the two projections

Species	Original range size	Predicted range size
	M/L	M/L
Reindeer (*Rangifer tarandus*)	18.1/18.0	18.0/18.1
Red deer (*Cervus elaphus*)	15.3/15.3	15.3/14.9
Siberian roe deer (*Capreolus pygargus*)	12.0/12.0	12.0/11.9
Eurasian elk / Moose (*Alces alces*)	7.8/7.9	7.8/8.2
Mule deer (*Odocoileus hemionus*)	6.3/6.3	6.3/6.5
Argali/Mountain sheep (*Ovis ammon*)*	3.5/3.5	3.5/3.1
Muskox (*Ovibos moschatus*)	1.8/1.7	1.8/1.6
Alpine musk deer (*Moschus chrysogaster*)*	1.3/1.3	1.3/1.2
Thinhorn sheep (*Ovis dalli*)	0.8/0.8	0.8/0.9
Mongolian gazelle (*Procapra gutturosa*)*	0.7/0.7	0.7/0.7
Bighorn sheep (*Ovis canadensis*)	0.5/0.5	0.5/0.5
Bactrian camel (*Camelus bactrianus*)*	0.2/0.2	0.2/0.2

Species range data were obtained as polygons from the IUCN Red List (IUCN 2014, downloaded 6 June 2014). These ranges represent large‐scale mammal distributions based on observations and expert reviews (Boitani et al. 2011). For the analyses, the unprojected original range polygons were projected to long‐lat and equal‐area grid, respectively, and every cell covered more than 10% by the range polygon was assigned a presence, otherwise an absence. Earlier investigations with a 50% threshold had shown this arbitrary value to be of little influence for the later results.

### Environmental variables

In a preliminary study, we used several variables as potential predictors across all species (climate, elevation, soil properties, land cover, net primary productivity). Four predictors were identified as important for all species: mean annual temperature, mean diurnal temperature range, total annual precipitation, and precipitation seasonality. All of those climate variables were obtained from the WorldClim dataset version 1.4 (Hijmans et al. 2005). They were downloaded as raster maps with a resolution of 10 arc‐minutes, corresponding to roughly 18.6 km at the equator. WorldClim variables are widely used in studies performing species distribution or ecological niche modeling (Synes and Osborne. 2011). For the purposes of this study, these four predictors were used in all species distribution models for maximal comparability among species. The highest variance inflation factor among the four selected predictors was 2.1; hence, we could safely assume no collinearity among predictors (Dormann et al. 2013).

### Spatial projections

The aim of this study was to analyze differences in model fits for the same response and predictors variables but with different map projections. We considered two basic types, a degree‐based long‐lat projection (L) and an equal‐area projection (Mollweide: M). For the long‐lat projection, we used WGS 84 coordinate reference system with a cell size of 1${}^{\circ }$. This particular degree projection is very popular due to its simplicity of using latitude and longitude as if they were Cartesian coordinates. The resolution of 1${}^{\circ }\times 1{}^{\circ }$ is equivalent to approximately 110×110 km${}^{2}$ at the equator. In this projection, latitudinal distances between each degree remain the same, whereas a degree of longitude is widest at the equator and gradually shrinks to zero at the poles (Fig. 2). For example, mid‐way to the poles, at 45${}^{\circ }$ North or South, the distance between a degree of longitude decreases to about 80 km.

For the equal area projection, we chose the Mollweide projection (M) with a 100×100 km${}^{2}$ cell size. This projection is occasionally used to visualize species distributions on subglobal extents while maintaining equal areas for the cells. As Fig. 1 illustrates, both latitude and longitude appear increasingly distorted when moving from the equator to the poles. This projection can be re‐centered to be viewed at any meridian, but the Africa‐centered projection is the most common for terrestrial applications.

### Geospatial transformation

**Figure 3:**
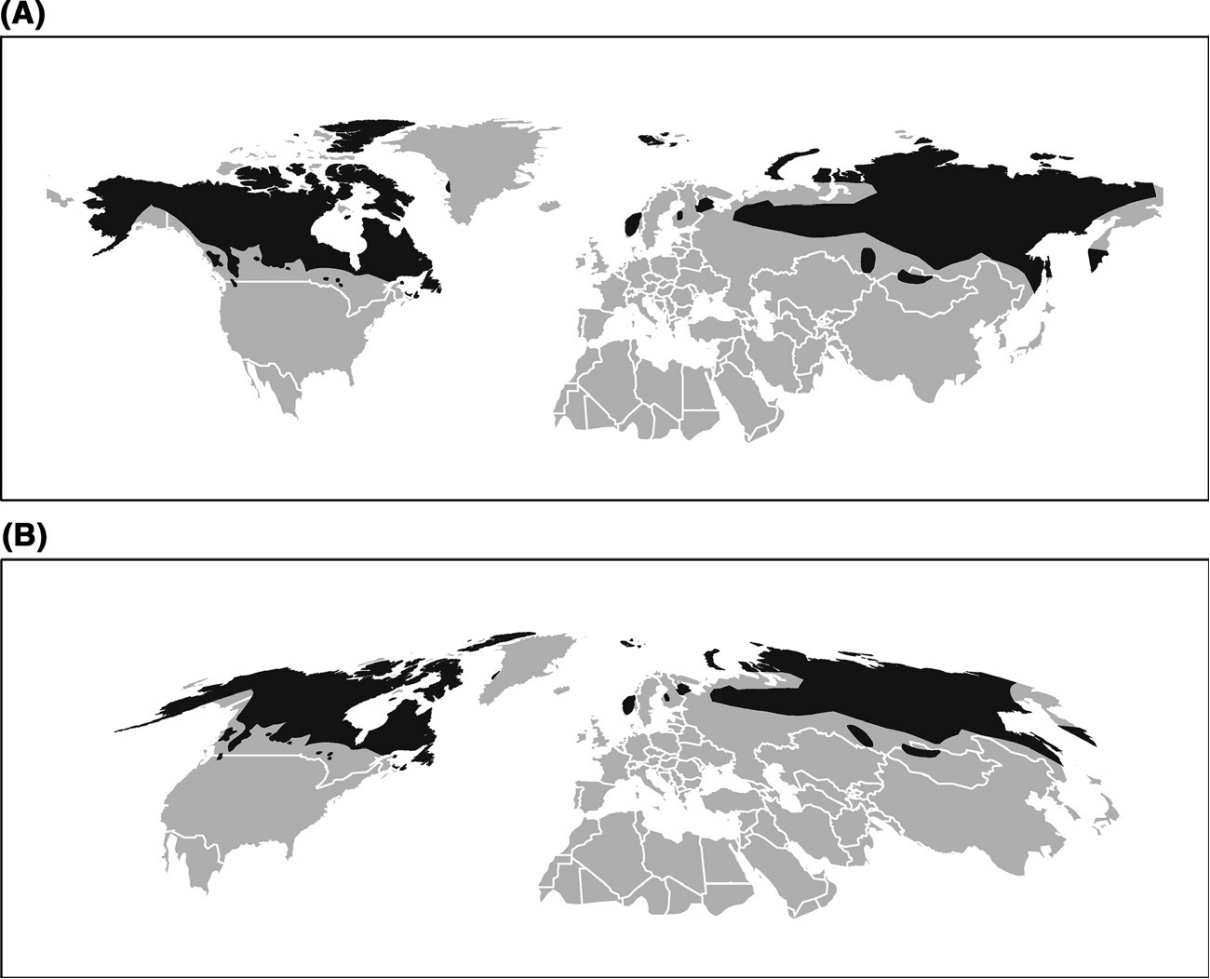
Distribution range of Reindeer (*Rangifer tarandus*) for long‐lat projection (left), and the Mollweide equal‐area projection (right). World map is clipped to the extent of the holarctic.

Both species range data and environmental data were available as unprojected long‐lat data, which is equivalent to an equidistant cylindrical projection.^1^ For equal‐area projection, the original data were transformed to Mollweide projection. All GIS operations and statistical analyses were carried out in R (R core Team 2014), primarily using the packages raster (Hijmans 2014) and sp (Bivand et al. 2013). Detailed R‐code is available on request from the last author. The final results of cutting the extent and re‐projecting the presence values of an example species can be seen in Fig. 3.

### Weighting Variables

Cell area was computed for each cell of the long‐lat projection and divided by maximum cell area. This value was used as weight for cell area.

The proportion of a cell covered by land was computed based on the Global Land Cover 2000 (Fritz et al. 2003). Layer covering water bodies was extracted for both projections separately and its inverse was then used as a weight for land cover.

For the model with both corrections, cell‐area, and land‐cover weights were multiplied.

### Model fitting

The ecological niche for each of the 12 species was estimated with a binomial generalized linear model (GLM). As the purpose of the study was to compare the effect of different projections, we did not aim to find the optimal model for each species. Therefore model quality will not be discussed in this paper.

For each species six model variants were computed (Table 2), each with a different set of rules: two “regular” models for each L and M dataset, and four models with an additional vector of weights used in the fitting process.

**Table 2:** Weighting schemes used for the six model variants. Each data point (representing a raster cell) was potentially weighted by cell area and/or land cover. L stands for long‐lat, M for the equal‐area Mollweide projection, A for area and L for land cover

Projection	Cell area	Land cover	Abbreviation
long‐lat	as is	as is	L
long‐lat	weighted	as is	LA
long‐lat	as is	weighted	LL
long‐lat	weighted	weighted	LLA
equal area	as is	as is	M
equal area	as is	weighted	ML

### Relative bias

To test for the effect of different projections and land cover on niche predictor estimates we calculated a “relative bias”. It is defined as the difference of parameter estimates between a model variant and a reference. We assume model variant ML to be closest to the statistical optimal solution (equal area, corrected for land cover) for our analysis and hence chose it as our reference. Thus we calculated for every parameter estimate *i* (except the intercept) the relative bias (*δ*) as an absolute standard score:

$$\delta_{i}^{D}=\left|\frac{\beta_{i}^{D}-\beta_{i}^{\mathrm{ref}}}{se_{\beta_{i}}^{\mathrm{ref}}}\right|,$$

where *β* is model coefficient, *D* the model variant, “ref” the reference model (ML) and *se* its standard error. This rescales all estimation biases to multiples of the standard error of the reference, making them comparable across predictors and species. In this calculation, the quality of the estimate fit has a strong influence on the outcome, because the better the estimation, the smaller the standard error, and hence, the larger is *δ*. Standard errors were similar for all model parameters among the reference and other model variants ($F_{5,66}<0.073$, *P* > 0.99 for all parameters), meaning the same parameters were estimated with approximately equal accuracy. Climatic datasets slightly differ among projections; as cell areas of long‐lat projection decrease toward the north, the number of cells concurrently increases, causing an overrepresentation of northern conditions compared to Mollweide projection. Standardizing variables with different means would yield different standardized model coefficients and larger bias estimates than with unstandardized predictors. Hence we kept all predictors unstandardized, but standardized model parameters after fitting the model.

The per‐parameter bias $\delta_{i}$ is then summarized for each species into one value per model by calculating a weighted arithmetic mean $\bar{\delta^{D}}$:

$$\bar{\delta^{D}}=\frac{\sum_{i}w_{i}\delta_{i}^{D}}{\sum_{i}w_{i}}$$

where $w_{i}$ is the partial explained deviance of predictor *i* in one of the four model variants *D*. Predictors with large influence, and hence large explained deviance, receive a large weight and therefore contribute most to the final values. This represents the idea that distortion in an important parameter has a stronger effect on the model than distortions in less influential parameters.

We analyzed relative bias using mixed effect models (Pinheiro et al. 2014) with “species” as random effect. Predictors indicate whether the range was entirely continental, maximum latitude of the range,  ln (range size), fit of the GLM model (pseudo‐$R^{2}$), land‐cover weighting (yes/no) cell‐area weighting (yes/no), and projection. To evaluate the effect of changes of parameter estimates, we computed the range area for each model. To do so, we multiplied the probability of occurrence with the cell area and summed all values. This avoids having to take an arbitrary threshold and is mathematically exact (Calabrese et al. 2014).

## Results

**Figure 4:**
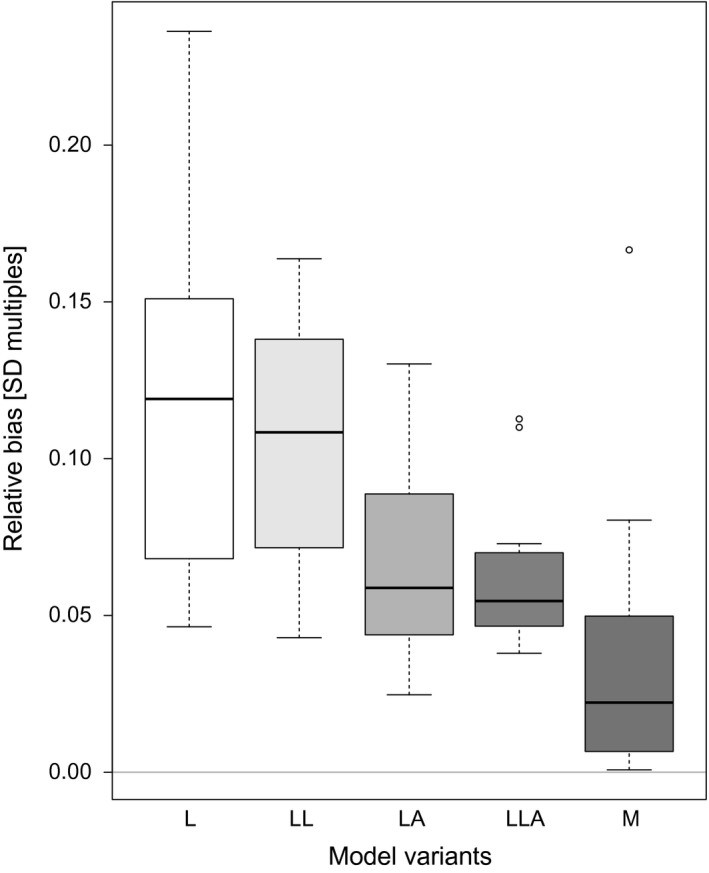
Relative parameter bias $\bar{\delta^{D}}$ across the GLMs of the 12 species. The zero‐line represents the Mollweide reference, corrected for land cover. A value of 0.1 indicates that parameters differed, on average, by 0.1 standard errors from model variant ML (see Table 2). The dark‐shaded model M indicates the effect of not correcting coastal cells for land coverage. Tukey's honest significant difference post hoc test identified differences between M and each of L, LA, LL, and LLA to be significant (*P* < 0.05).

Analyzing bias as measured by $\bar{\delta^{D}}$, we found a limited but significant difference between our reference model ($F_{4,55}=10.82$, *P* < 0.001), the land‐cover corrected Mollweide projection (ML) and all other model variants (Fig. 4). Differences due to projections and the cell‐area correction were the strongest contributors to variation in $\bar{\delta^{D}}$ (Table 3). The mean relative bias (Fig. 4 displays medians) for the model L was 0.12, for LA 0.07, for LL 0.11, for LLA 0.06, and for M 0.04. Including land cover reduced the bias in the conformal projection marginally, and including area correction or both, substantially. Bias was lowered for LLA, but not completely eliminated, suggesting that there is an intrinsic difference between the projections that could not be corrected by weighting.

**Table 3:** Mixed effect model analysis of factors influencing relative bias, with species as random factor. All other first‐ and second‐order interactions were not significant and hence removed from the final model

	numDF	denDF	*F*‐value	*P*‐value
(Intercept)	1	44	1566.91	< 0.0001
log (range size)	1	8	1.54	0.25
maximum latitude	1	8	13.89	0.0058
projection	1	44	43.00	< 0.0001
area weighting	1	44	13.87	0.0006
land‐cover weighting:coastal	1	44	3.39	0.0723

**Figure 5:**
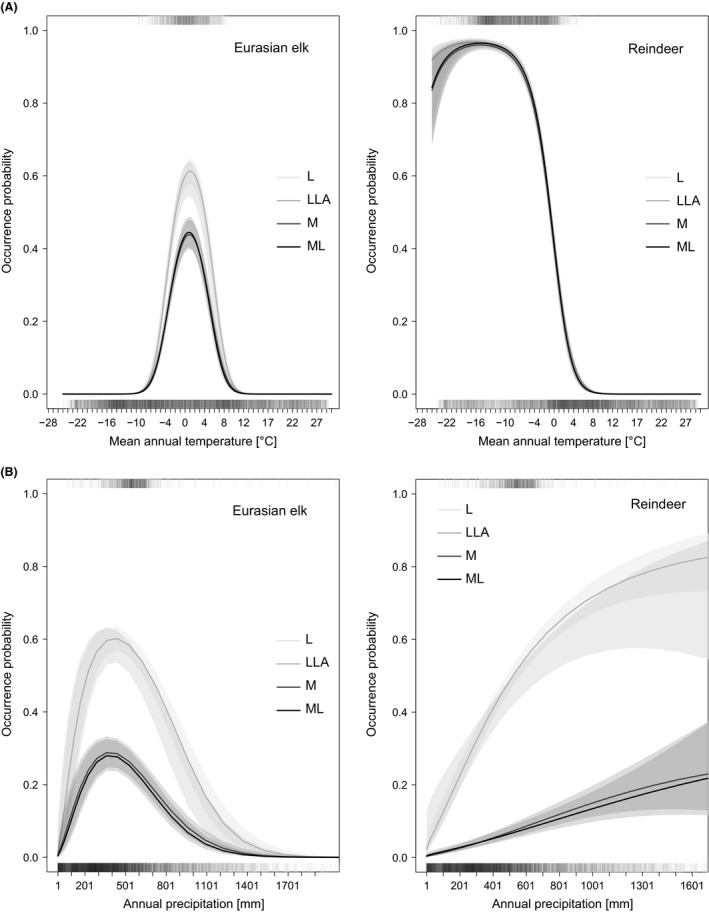
Climate response curves of Eurasian elk (left) and reindeer (right) with respect to temperature (top) and precipitation (bottom) from four different model variants. To accommodate the skewness in precipitation, the *x*‐axis was cut off at 99% of the data. Overlapping 95%‐confidence intervals create heavier shading.

Parameter bias needs not result in dramatic differences in the occurrence‐environment relationship, as, for example, linear and quadratic terms are correlated and can to some extent compensate for changes in the other parameter. For reindeer and Eurasian elk, the species with the largest relative bias in their group, estimation bias had an impact on occurrence probability level, but did not translate into very different forms of climate response curves, and model predictions from any of the model variants we trialed is thus likely to be very similar (Fig. 5).

## Discussion

Geographical projection, in particular the choice between conformal and equal‐area projections, is likely to affect the estimation of the effect of environmental predictors of species' distributions. We showed a strong effect of correcting for the area of the cell, which seems to be the main effect when changing between the two projections (Fig. 4).

**Figure 6:**
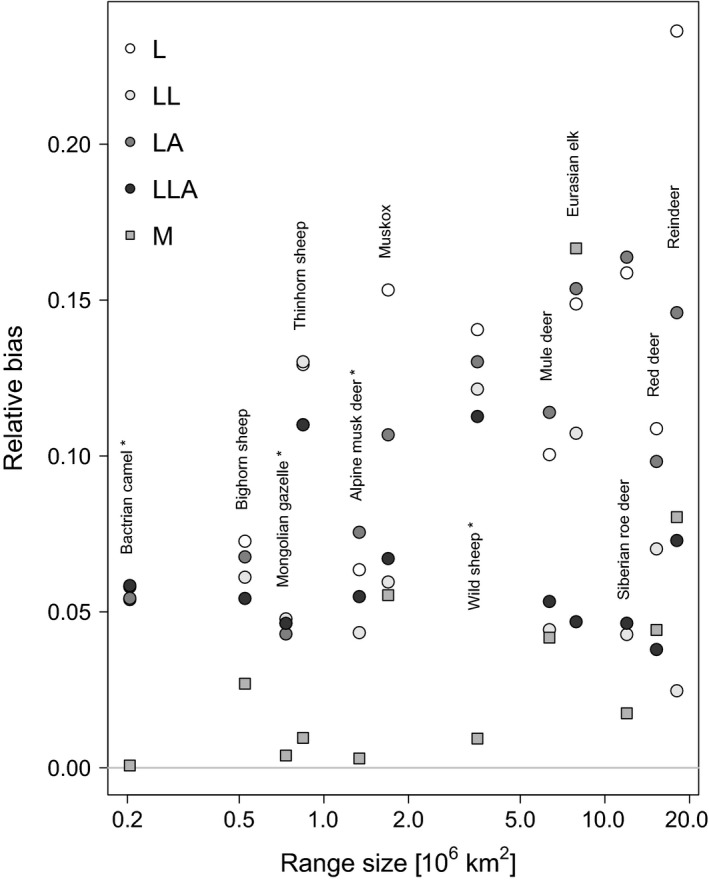
Relative parameter bias $\bar{\delta^{D}}$ for each model, as a function of species range size. Asterisks behind species names indicate continental species. The increase in bias with log(range size) is significant, as is the difference between projections (circles vs. squares).

We have purposefully selected 12 species with high‐latitudinal distribution, as the difference in cell area between conformal and equal‐area projections increases toward the poles (Fig. 2). Species with more temperate to tropical distributions will accordingly show substantially less projection‐induced bias. But even in these worst‐case scenarios, and despite a detectable effect on model parameter estimates, we conclude that such a low bias will affect neither inferential statistical analyses nor predictions of species occurrence probabilities to any noteworthy amount. Estimates always come with a certain uncertainty, and although 95%‐confidence intervals of predictions of the most extreme model variants might not overlap for all predictor variables, this seem largely attributable to divergent predicted probabilities rather than different functional relationships. Secondly, we showed that the main difference between projections stem from having cells of different sizes, and that this effect could be significantly reduced by weighting each cell for its area (Fig. 4). Finally, the often overlooked effect of weighting data points by the proportion of cell covered by land further reduced the bias, but its effect was less severe. The focus of our study was the projection effect, and hence, the land‐cover effect has not been as rigorously tested as it could be, for example, using coastal or island species. Still, its effect can be seen in the comparison of purely continental species (marked by an asterisk in Table 1 and Fig. 6). The impact of land cover was particularly pronounced in species with large range size (>5 million km${}^{2}$). The least biased model was the only variant based on the same projection as our reference model (M), confirming that there are intrinsic differences between the two projections which could not be removed by correcting for land cover and cell area (Fig. 6).

### Potential effects of changing cell area

The decreasing cell area toward the poles in conformal projections may affect model quality in at least four ways. First, the environmental conditions of that cell become more homogeneous. The smaller the cell, the less different environmental conditions are encountered and hence averaged over. Thus, moving to smaller cells at the poles should reduce subcell variability. Statistically, this is an interesting effect, as in smaller cells the uncertainty of the true conditions experienced by the focal species decreases, that is, we have less measurement error in our predictor values. This in turn reduces the bias on the slope estimates due to regression dilution (Madansky 1959; Frost and Thompson 2000), a phenomenon not much discussed in ecology (McInerny and Purves 2011; Calabrese et al. 2014), but common in medical statistics (Fuller 1987; Knuiman et al. 1998). It could thus be that lat‐long projected data experience slightly less regression dilution and hence exhibit slightly higher absolute parameter estimates. We did observe regression dilution in our analyses, albeit only for two of eight predictors. We thus tentatively conclude that the increase in accuracy of environmental data due to smaller cell size did not play an important role in our study.

Second, (pointed out by Petr Keil in the reviewing process) the fact that the importance of environmental predictors in the model changes with grain size could also have an impact on parameter estimates. Climate predictors are usually more important toward coarse grain analyses, while habitat characteristics come to bear in fine grains (e.g.,Luoto et al. 2007). As cell size substantially decreases with latitude, long‐lat projections could thus give more weight to large‐scale important predictors toward the equator and fine‐grain predictors toward the poles. More generally, the difference in cell size among the two projections can lead to slightly different models. Partial R${}^{2}$ differed more between models weighting for cell area (LA, LLA, M, ML) or not (L, LL), rather than between the two projections (three of four predictors at 0.05 level, and 1 predictor respectively). Apparently cell‐size correction was able to compensate the underlying differences among projections in this case. As predictors with large influence contribute most to the bias estimate, this might have an impact for the results.

Third, the probability of observing a species in a cell increases as the area of that cell increases, all else being equal. As our data are based on expert‐drawn ranges, rather than real observations in each cell, we would expect larger cells to be slightly more accurate when claiming a species to be present (Graham and Hijmans. 2006; Pineda and Lobo 2012). Moreover, the area covered by a smaller cell represents a smaller portion of species range, hence smaller cells should be down‐weighted, which is what we did in our model variant LA (lat‐long, area‐adjusted). Differences between L (long‐lat) and LA were small but relatively consistent across the 12 species (Figs 4 and 6). We conclude that this effect of projection also had an effect on parameter estimates.

Fourth, changing the projection may change the actual number of data points, unless we tune the cell size of the equal‐area projection to exactly match the number of cells. In our case, moving from the 100 × 100 km grid to the 1 × 1${}^{\circ }$ grid shrunk the mean cell area from 10,000 to 7229 km${}^{2}$. Alongside, the number of terrestrial cells increased from 7966 to 11,018 . Without systematic analysis it is difficult to guess what the effect of this change may be, but the analysis of Wisz et al. 2008 suggests that sample size effects on model quality level off. For the most common species, reindeer, changes in prevalences (from 23% to 30%) were the largest and changes in estimates the second largest among our study species , suggesting that the increase in the number of data points may be a contributor to relative bias. Clearly our analysis is not elaborated enough to speak decisively on the effect of changing the number of data points.

### Effects of proportion of land in a cell

The inclusion, or not, of a weight for the proportion of a cell actually covered by land had a lower effect compared to the change of cell area and projection. For species with ranges up to the coast, coastal cells may be filled only to a small fraction by land. While we computed climatic conditions based only for the land‐covered part of the cell (masking the sea), such cells may actually be particularly vulnerable to inaccurate presence or absence data. Often range maps are simply extended up to the coast, suggesting a species may occur on stretch of land reaching into a new cell, while in fact the species has never been observed there. Coastal cell are, for example, more densely populated than inland cells, making them less attractive for large mammals. Granting a cell with only a small fraction being filled by land the same weight in the analysis as a full‐covered continental cell ignores the higher likelihood of a species' presence being an error (as argued in the previous section).

Also this effect requires to be investigated more deeply, and with data providing direct evidence for species presence, as to our knowledge no weighting for total land cover is being applied in species distribution analyses. In fact, we did not find a single study that considered this effect or mentioned its potential relevance (admittedly this is difficult to search the literature for), although weighting of data points is no new idea (e.g., Broennimann et al. 2006; Maggini et al. 2006; Graham et al. 2008).

## Conclusion

Overall, our findings are good news for studies that were carried out using long‐lat projections at large spatial scales. One limitation of our study is that we did not investigate the projection effect at small cell sizes, such as used in studies of smaller extent. However, small‐cell, long‐lat projection studies are very rare, as topographic, infrastructural, and environmental data at country or regional level are often provided in equal‐area projections. At such extents angular distortions, which are the main argument in favor of long‐lat projections, are less severe. In fact, all small‐scale studies we found were carried out on equal‐area projections, such as the European Environmental Agencies reference grid.^2^

In conclusion, we recommend generally using equal‐area projections whenever analyzing species distributions. Moreover, we strongly encourage the analyst to consider weighting cells proportionally to their size, as a small cell contains information about a smaller part of the species range compared to a more inclusive large cell. Likewise, the reliability of information in a cell should be accounted for in the same manner. In our case, we deemed cells only partly covered by land as less reliable sources of presence of a species, but the same approach can obviously be used for measures of sampling intensity and alike. Also is the use of weights not restricted to the GLMs we used here. Most flexible algorithms, both parametric and machine‐learning, can accommodate case‐specific weights.

## Conflict of Interest

None declared.

## Supporting information


**Data S1.** The complete R‐code used for data analysis

## Notes

1
http://www.remotesensing.org/geotiff/proj_list/equirectangular.html

2
http://www.eea.europa.eu/data-and-maps/data/eea-reference-grids-1

## References

[ece31838-bib-0034] Bivand, R. S. , E. Pebesma , and V. Gomez‐Rubio . 2013 Applied Spatial Data Analysis with R, 2nd edn. Springer, New York.

[ece31838-bib-0001] Bled, F. , J. D. Nichols , and R. Altwegg. 2013 Dynamic occupancy models for analyzing species' range dynamics acress large geographic scales. Ecol. Evol. 3:4896–4909. 24455124 10.1002/ece3.858 PMC3892356

[ece31838-bib-0002] Boitani, L. , L. Maiorano , D. Baisero , A. Falcucci , P. Visconti , and C. Rondinini 2011 What spatial data do we need to develop global mammal conservation strategies?. Philos. Trans. R. Soc. B Biol. Sci. 366:2623–2632. 10.1098/rstb.2011.0117 PMC3140738 21844041

[ece31838-bib-0003] Bonn, A. , D. Storch , and K. J. Gaston. 2004 Structure of the species–energy relationship. Proc. R. Soc. B Biol. Sci. 271:1685–1691. 10.1098/rspb.2004.2745 PMC1691779 15306288

[ece31838-bib-0004] Botts, E. A. , B. F. N. Erasmus , and G. J. Alexander. 2013 Small range size and narrow niche breadth predict range contractions in South African frogs. Glob. Ecol. Biogeogr. 22:567–576.

[ece31838-bib-0005] Broennimann, O. , W. Thuiller , G. Hughes , G. F. Midgley , J.M. R. Alkemade , and Guisan A. 2006 Do geographic distribution niche property and life form explain plants' vulnerability to global change? Glob. Chang. Biolo. 12:1079–1093.

[ece31838-bib-0006] Calabrese, J. M. , G. Certain , C. Kraan , and C. F. Dormann. 2014 Stacking species distribution models and adjusting bias by linking them to macroecological models. Glob. Ecol. Biogeogr. 23:99–112.

[ece31838-bib-0007] Cumming, G.S. , and S. P. Road. 2000 Using between‐model comparisons to fine‐tune linear models of species ranges. J. Biogeogr. 27:441–445.

[ece31838-bib-0008] Dormann, C. F. , J. Elith , S. Bacher , C. M. Buchmann , G. Carl , G. Carre , J. R. Garcia Marquez , B. Gruber , B. Lafourcade , P. J. Leitao , et al. 2013 Collinearity: a review of methods to deal with it and a simulation study evaluating their performance. Ecography, 36:27–46.

[ece31838-bib-0009] Fritz, S. , E. Bartholome , A. Belward , A. Hartley , H.‐J. Stibig , H. Eva , P. Mayaux , S. Bartalev , R. Latifovic , S. Kolmert , et al. 2003 Harmonisation mosaicing and production of the Global Land Cover 2000 database (Beta Version).

[ece31838-bib-0010] Frost, C. , and S. G. Thompson. 2000 Correcting for regression dilution bias: comparison of methods for a single predictor variable. J. R. Stat. Soc. A 163:173–189.

[ece31838-bib-0011] Fuller, W. A. 1987 Measurement Error Models. Wiley, New York.

[ece31838-bib-0012] Gaston, K. J. , R. G. Davies , C. D. L. Orme , V. A. Olson , G. H. Thomas , T.‐S. Ding , P. C. Rasmussen , J. J. Lennon , P. M. Bennett , I. P. Owens , et al. 2007 Spatial turnover in the global avifauna. Proc. R. Soc. B Biol. Sci. 274:1567–1574. 10.1098/rspb.2007.0236 PMC2169276 17472910

[ece31838-bib-0013] Graham, C. H. , and R. J. Hijmans. 2006 A comparison of methods for mapping species ranges and species richness. Glob. Ecol. Biogeogr. 15:578–587.

[ece31838-bib-0014] Graham, C. H. , J. Elith , R. J. Hijmans , A. Guisan , P. A. Townsend , B. A. Loiselle , and the NCEAS Predicting Species Distributions Working Group . 2008 The influence of spatial errors in species occurrence data used in distribution models. J. Appl. Ecol. 45: 239–247.

[ece31838-bib-0015] Gudmundsson, G. A. , and T. Alerstam . 1998 Optimal map projections for analysing long‐distance migration routes. J. Avian Biol. 29: 597–605.

[ece31838-bib-0016] Gwitira, I. , A. Murwira , M. D. Shekede , M. Masocha , and C. Chapano. 2013 Precipitation of the warmest quarter and temperature of the warmest month are key to understanding the effect of climate change on plant species diversity in Southern African savannah. Afr. J. Ecol. 52:–216.

[ece31838-bib-0017] Hartley, S. , R. Harris , and P. Lester . 2006 Quantifying uncertainty in the potential distribution of an invasive species: climate and the Argentine ant. Ecol. Lett. 9: 1068–1079. 16925656 10.1111/j.1461-0248.2006.00954.x

[ece31838-bib-0033] Hijmans, R. J. 2014 *raster: Geographic data analysis and modeling* . R package version 2.2‐31.

[ece31838-bib-0018] Hijmans, R. J. , S. E. Cameron , J. L. Parra , P. G. Jones , and A. Jarvis. 2005 Very high resolution interpolated climate surfaces for global land areas. Int. J. Climatol. 15:1965–1978.

[ece31838-bib-0019] Holmgren, M. , and L. Poorter. 2007 Does a ruderal strategy dominate the endemic flora of the West African forests?. J. Biogeogr. 34:1100–1111.

[ece31838-bib-0020] IUCN . 2014 The IUCN Red List of threatened species. Version 2014.1. #http://www.iucnredlist.org #

[ece31838-bib-0022] Keil, P. , and B. A. Hawkins. 2009 Grids versus regional species lists: are broad‐scale patterns of species richness robust to the violation of constant grain size. Biodivers. Conserv. 18:3127–3137.

[ece31838-bib-0023] Knuiman, M. W. , M. L. Divitini , J. S. Buzas , and P. E. B. Fitzgerald. 1998 Adjustment for regression dilution in epidemiological regression analyses. Ann. Epidemi. 8:56–63. 10.1016/s1047-2797(97)00107-5 9465995

[ece31838-bib-0024] Kriticos, D. J. , and A. Leriche. 2010 The effects of climate data precision on fitting and projecting species niche models. Ecography 33:115–127.

[ece31838-bib-0025] Lovett, J. O. N. C. , S. Rudd , and J. Taplin. 2000 Patterns of plant diversity in Africa south of the Sahara and their implications for conservation management. Biodivers. Conserv. 9:37–46.

[ece31838-bib-0026] Luoto, M. , R. Virkkala , and R. K. Heikkinen. 2007 The role of land cover in bioclimatic models depends on spatial resolution. Glob. Ecol. Biogeogr. 16:34–42.

[ece31838-bib-0027] Madansky, A. 1959 The fitting of straight lines when both variables are subject to error. J. Am. Stat. Assoc. 54:173–205.

[ece31838-bib-0028] Maggini, R. , A. Lehmann , N. E. Zimmermann , and A. Guisan. 2006 Improving generalized regression analysis for the spatial prediction of forest communities. J. Biogeogr. 33:1729–1749.

[ece31838-bib-0029] McInerny, G. J. , and D. W. Purves. 2011 Fine‐scale environmental variation in species distribution modelling: regression dilution latent variables and neighbourly advice. Methods Ecol. Evol. 2:248–257.

[ece31838-bib-0030] Mulcahy, K. A. , and K. C. Clarke. 2001 Symbolization of map projection distortion: A review. Cartogr. Geogr. Inf. Sci. 28:167–182.

[ece31838-bib-0036] Peterson, A. T. , J. Soberon , R. G. Pearson , R. P. Anderson , E. Martinez‐Meyer , M. Nakamura , and M. B. Araujo . 2011 Ecological Niches and Geographic Distributions. Princeton University Press, Princeton NJ.

[ece31838-bib-0031] Pineda, E. , and J. M. Lobo. 2012 The performance of range maps and species distribution models representing the geographic variation of species richness at different resolutions. Glob. Ecol. Biogeogr. 21:935–944.

[ece31838-bib-0021] Pinheiro, J. , D. Bates , S. DebRoy , D. Sarkar , R Core Team . 2014 *nlme: Linear and Nonlinear Mixed Effects Models* . R package version 3.1‐118.

[ece31838-bib-0032] R Core Team . 2014 *R: A Language and Environment for Statistical Computing* . R Foundation for Statistical Computing. http://www.R-project.org, Vienna Austria.

[ece31838-bib-0038] van Rensburg, B. J. , S. L. Chown , and K. J. Gaston. 2002 Species richness environmental correlates and spatial scale: a test using South African birds. Am. Nat. 159:566–577. 18707437 10.1086/339464

[ece31838-bib-0035] Synes, N. W. , and P. E. Osborne . 2011 Choice of predictor variables as a source of uncertainty in continental‐scale species distribution modelling under climate change. Glob. Ecol. Biogeogr. 20:904–914.

[ece31838-bib-0037] UNEP‐WCMC . 2004 Biogeographic Realms (Generalised)#http://www.arcgis.com/home/item. #html?27eef65481234036bdf55a78150e1f9d. accessed: 19.10.2013.

[ece31838-bib-0039] Veloz, S. D. , J. W. Williams , J. L. Blois , F. He , B. Otto‐Bliesner , and Z. Liu. 2012 No‐analog climates and shifting realized niches during the late quaternary: implications for 21st‐century predictions by species distribution models. Glob. Chang. Biol. 18:1698–1713.

[ece31838-bib-0040] Wisz, M. S. , R. J. Hijmans , J. Li , A. T. Peterson , C. H. Graham , A. Guisan , and the NCEAS Predicting Species Distributions Working Group . 2008 Effects of sample size on the performance of species distribution models. Divers. Distrib. 14: 763–773.

