# Supplementary material for: Squares of different sizes: effect of geographical projection on model parameter estimates in species distribution modeling
Source: Ecol Evol. 2015 Dec 17;6(1):202–11. doi: 10.1002/ece3.1838 (PMC4716504; doi:10.1002/ece3.1838)
Supplement: Supplementary file 1 — Data S1. The complete R‐code used for data analysis [file ECE3-6-202-s001.pdf]

```
# Contents
# 1. extracting ungulates to 1x1 degree and 100x100 km grid
# 2. Extracting environmental variables
# 3. Running GLM species distribution models for the two projection types
# 4. Calculating relative bias in parameter estimates among model variants

library(raster)
library(rgdal)
library(maptools)
library(lattice)

# Load IUCN shape file
mams <- readShapeSpatial("/.../TERRESTRIAL_MAMMALS") # unprojected,
proj4string(mams)<- CRS("+proj=longlat +datum=WGS84") # the datum (wgs84) is written in metadata and added manually

# Function to extract species
extract.species <- function(mams, species){
  # SPDF is the spatialPolygonsDataFrame object
  # species.name of the species to be extracted
  # author: Carsten F. Dormann
  if ("binomial" %in% names(mams@data))  ind <- which(mams@data$binomial == species)
  selected.species <- mams
  selected.species@data <- mams@data[ind,]
  selected.species@polygons <- mams@polygons[ind]
  selected.species@plotOrder <- seq_along(ind)
  return(selected.species)
}

# Ungulates to extract
species <- c("Moschus chrysogaster", "Odocoileus hemionus", "Capreolus pygargus", "Procapra gutturosa", "Camelus ferus", "Ovis ammon", "Ovis dalli", "Ovis canadensis", "Cervus elaphus", "Alces alces", "Rangifer tarandus", "Ovibos moschatus")


## -- Extract species to 1 degree grid -- ##

ll <- raster(mams) # Crate an empty raster with 1 degree resolution
res(ll) <-1 # set the resolution to 1 degree
save(ll, file="empty_raster_1deg_iucn_extent.Rdata")

raster.ungulates <- rasterize(extract.species(mams, species[1]), ll)
for (i in 2:length(species)){
  raster.ungulates2 <- rasterize(extract.species(mams, species[i]), ll)
  raster.ungulates <- addLayer(raster.ungulates, raster.ungulates2)
  print(paste("Finished the ", i, "th species: ", species[i], sep=""))
}

species <- gsub(" ","_",species)
names(raster.ungulates) <- species

## -- Eliminating any non-Holarctic ranges -- #

# The extent of the raster remain the same, but any values outside the holarctic polygon will be turned to NA
# source http://www.arcgis.com/home/item.html?id=27eef65481234036bdf55a78150e1f9d
setwd(".../gen_bio_realm")
bio_realms <- readShapeSpatial("gen_bio_realm_2004.shp") # GEt the shape of Holarctic
holarctic <- SpatialPolygons(bio_realms@polygons[c(1,2,3,4)])
proj4string(holarctic)<- CRS("+proj=longlat +datum=WGS84") # set the coordinate system

# from the suggestion (https://stat.ethz.ch/pipermail/r-sig-geo/2012-June/015260.html)
crop  <- setValues(raster.ungulates, NA) # Create a dummy NA raster with a spatial extension equal to the cropped raster
myshp.r <- rasterize(holarctic, crop) #  Rasterize the catchment boundaries, with NA outside the catchment boundaries
ungulates.hol <- mask(x=raster.ungulates, mask=myshp.r)
plot(ungulates.hol$Rangifer_tarandus)
plot(holarctic, add=T)

# extract the coordinates and save in a dataframe format
coord <- as.data.frame(xyFromCell(ungulates.hol, 1:ncell(ungulates.hol))) # extract the coordinates.
ungulates_cover_1deg <- cbind.data.frame(coord, ungulates = values(ungulates.hol)) # bind them to the occurrence data
colnames(ungulates_cover_1deg) <- gsub("ungulates.","",colnames(ungulates_cover_1deg))
ungulates_cover_1deg$ID <- paste(ungulates_cover_1deg$x, ungulates_cover_1deg$y, sep="+")

save(ungulates_cover_1deg, file="ungulates_cover_1degree.Rdata")


## -- Extracting ungulates to 100 x 100 Mollweide -- ##

# The same as before, just with different projection
# Transform IUCN data to mollweide equal area projection
mams <- spTransform(mams, CRS('+proj=moll'))
```

```
# Create an empty raster in equal area projection with 100 x 100 km reslution and the same extent as mammals range polygons
moll <- raster(mams)
res(moll) <- 100000 # set the resolution
save(moll, file = "empty_raster_100km_iucn_extent.Rdata")

species <- gsub("_"," ",species)

raster.ungulates <- rasterize(extract.species(mams, species[1]), moll)
for (i in 2:length(species)){
  raster.ungulates2 <- rasterize(extract.species(mams, species[i]), moll)
  raster.ungulates <- addLayer(raster.ungulates, raster.ungulates2)
  print(paste("Finished the ", i, "th species: ", species[i], sep=""))
}

species <- gsub(" ","_",species)
names(raster.ungulates) <- species

# Remove non-holarctic ranges
holarctic_moll <- spTransform(holarctic, "+proj=moll") # set the Mollweide projection to holarctic
crop  <- setValues(raster.ungulates, NA)
myshp.r <- rasterize(holarctic_moll, crop)
ungulates.hol <- mask(x=raster.ungulates, mask=myshp.r)
plot(ungulates.hol$Rangifer_tarandus)
plot(holarctic_moll, add=T)

# extract coordinates and save in a dataframe format.
coord <- as.data.frame(xyFromCell(ungulates.hol, 1:ncell(ungulates.hol)))
ungulates_cover_Moll_100 <- cbind.data.frame(coord, ungulates =values(ungulates.hol))
colnames(ungulates_cover_Moll_100) <- gsub("ungulates.","",colnames(ungulates_cover_Moll_100))
ungulates_cover_Moll_100$ID <- paste(ungulates_cover_Moll_100$x, ungulates_cover_Moll_100$y, sep="+")

levelplot(Rangifer_tarandus ~x+y,data=ungulates_cover_Moll_100) # test plot
save(ungulates_cover_Moll_100, file="ungulates_cover_Mollweide_100.Rdata")


                    ######################################################
                           ### --  Extracting environmental data -- ###
                    ######################################################

## -- Bioclim 1 deg -- ##

# Load Biolicm data
setwd(".../Raw_data")
bioclim.data <- getData('worldclim', var='bio', res=10, download=T) # 27.02.2015

# It is a rasterstack, CRS defined
# Aggregate to coarster resolution (1 deg). Water bodies have NA values so averaging will give correct outcome

bioclim <- projectRaster(from = bioclim.data, to=ll)
area <- area(ll) # Get the area of each cell in long-lat projection
names(area) <- "cell_area"
bioclim <- addLayer(bioclim, area)

# cut out non-holarctic areas
crop  <- setValues(bioclim, NA)
myshp.r <- rasterize(holarctic, crop)
bioclim.hol <- mask(x=bioclim, mask=myshp.r)

plot(bioclim.hol$cell_area)
plot(holarctic, add=T)

# Extracting the coordinates and saving in a dataframe.
coord <- as.data.frame(xyFromCell(bioclim.hol, 1:ncell(bioclim.hol)))
bioclim_cover <- cbind.data.frame(coord, bioclim =values(bioclim.hol))

colnames(bioclim_cover) <- gsub("bioclim.","",colnames(bioclim_cover))
bioclim_cover$ID <- paste(bioclim_cover$x, bioclim_cover$y, sep="+")

levelplot(bio1 ~x+y,data=bioclim_cover) # check how it looks
save(bioclim_cover, file="bioclim_cover.grid_1deg_holarctic.Rdata")


## -- Bioclim 100x100 Mollweide -- ##

# Change projection
bioclim <- projectRaster(from = bioclim.data, to=moll)

# Cut to holarctic
```

```
crop  <- setValues(bioclim, NA)
myshp.r <- rasterize(holarctic_moll, crop)
bioclim.hol <- mask(x=bioclim, mask=myshp.r)

plot(bioclim.hol$bio1)
plot(holarctic_moll, add=T)

# Extracting the coordinates and saving in a dataframe.
coord <- as.data.frame(xyFromCell(bioclim.hol, 1:ncell(bioclim.hol)))  # this extracts the coordinates.
bioclim_cover <- cbind.data.frame(coord, bioclim =values(bioclim.hol))
colnames(bioclim_cover) <- gsub("bioclim.","",colnames(bioclim_cover))
bioclim_cover$ID <- paste(bioclim_cover$x, bioclim_cover$y, sep="+")

save(bioclim_cover, file="bioclim_cover.grid_Moll100_holarctic.Rdata")


## -- Global land cover 1 degree -- ##

# Get GLC data
setwd(".../GLC2000_Tiff")
glc<-raster("glc2000_v1_1.tif")
load("~/new_IUCN/New_globaldataset/projections/empty_raster_1deg_iucn_extent.Rdata") # Load the empty raster

glcTerr <- glc ==20 # Terrestrial cover, original data is binary (0,1)

# To aggregate Terrestrial cover to coarser resolution, cells covered by water SHOULD NOT be turned to NA
# We need to know the percentage of land in a cell

glcTerrCoarse <- aggregate(glcTerr, fact=16, fun=mean, expand=TRUE, na.rm=TRUE)
values(glcTerrCoarse) <- (1-values(glcTerrCoarse))/1 # swap water and land values.
plot(glcTerrCoarse,xlim=c(-30,30),ylim=c(-20,20)) # perfect
projection(glcTerrCoarse) <- CRS("+proj=longlat +ellps=WGS84 +datum=WGS84 +no_defs +towgs84=0,0,0") # set the coordinate system
glcTerr_grid1 <- projectRaster(glcTerrCoarse, ll)
plot(glcTerr_grid1,xlim=c(-30,30),ylim=c(-20,20))
save(glcTerr_grid1, file= "landcover_grid1.Rdata")

# Cut to holarctic area
crop  <- setValues(glcTerr_grid1, NA) # Dummy raster with a spatial extension equal to the cropped raster,but full of NA values
myshp.r <- rasterize(holarctic, crop) #  Rasterize the catchment boundaries, with NA outside the catchment boundaries
glcTerr_grid1.hol <- mask(x=glcTerr_grid1, mask=myshp.r)
plot(glcTerr_grid1.hol$layer) # check how it looks

# Extracting coordinates and saving in data frame.
coord <- as.data.frame(xyFromCell(glcTerr_grid1.hol, 1:ncell(glcTerr_grid1.hol)))
Terr_cover_grid1 <- cbind.data.frame(coord, Landcover =values(glcTerr_grid1.hol))
Terr_cover_grid1$ID <- paste(Terr_cover_grid1$x, Terr_cover_grid1$y, sep="+")
save(Terr_cover_grid1, file=".../terrestrialCoverFromGLC20_grid1deg_holarctic.Rdata")


## -- Terrestrial cover 100 x 100 – ##

glcTerr_grid100 <- projectRaster(glcTerrCoarse, moll)
plot(glcTerr_grid100,xlim=c(-3000000,3000000),ylim=c(-2000000,2000000)) # Looks good!
save(glcTerr_grid100, file= "landcover_grid100.Rdata")


crop  <- setValues(glcTerr_grid100, NA) # Dummy raster with a spatial extension equal to the cropped raster,but full of NA values
myshp.r <- rasterize(holarctic_moll, crop) #  Rasterize the catchment boundaries, with NA outside the catchment boundaries

# Assigning NA values to all  raster cells outside holarctic shapefile boundaries
glcTerr_grid100.hol <- mask(x=glcTerr_grid100, mask=myshp.r)
plot(glcTerr_grid100.hol$layer) # check how it looks

# Extracting coordinates and saving in data frame.
coord <- as.data.frame(xyFromCell(glcTerr_grid100.hol, 1:ncell(glcTerr_grid100.hol)))
Terr_cover_grid100 <- cbind.data.frame(coord, Landcover =values(glcTerr_grid100.hol))
Terr_cover_grid100$ID <- paste(Terr_cover_grid100$x, Terr_cover_grid100$y, sep="+")
save(Terr_cover_grid100, file=".../terrestrialCoverFromGLC20_grid100_holarctic.Rdata")
levelplot(Landcover~ x + y, data = Terr_cover_grid100)



###   Merging environmental data ###

load("~/new_IUCN/New_globaldataset/projections/bioclim_cover.grid_Moll100_holarctic.Rdata")
load("~/new_IUCN/New_globaldataset/projections/ungulates_cover_Mollweide_100.Rdata")
load("~/new_IUCN/New_globaldataset/projections/terrestrialCoverFromGLC20_grid100_holarctic.Rdata")
list.of.data.frames100 = list(bioclim_cover[,c("x","y","bio1", "bio2", "bio12", "bio15", "ID")],
                         Terr_cover_grid100[,c("Landcover","ID")],
```

```
                              ungulates_cover_Moll_100[,3:15])

merged.global.dataset100 = Reduce(function(...) merge(..., all=T, by="ID"), list.of.data.frames100)
merged.global.dataset100[which(merged.global.dataset100$Landcover==0),c(4:20)] <- NA
save(merged.global.dataset100, file= "merged.global.dataset.100.Rdata")


######################################
load("~/new_IUCN/New_globaldataset/projections/bioclim_cover.grid_1deg_holarctic.Rdata")
load("~/new_IUCN/New_globaldataset/projections/ungulates_cover_1degree.Rdata")
load("~/new_IUCN/New_globaldataset/projections/terrestrialCoverFromGLC20_grid1deg_holarctic.Rdata")
list.of.data.frames1 = list(bioclim_cover[,c("x", "y", "bio1", "bio2", "bio12", "bio15", "cell_area", "ID")],
                            Terr_cover_grid1[,c("Landcover","ID")],
                            ungulates_cover_1deg[,3:15])

merged.global.dataset1 = Reduce(function(...) merge(..., all=T, by="ID"), list.of.data.frames1)
merged.global.dataset1[which(merged.global.dataset1$Landcover==0),c(4:8,9:21)] <- NA
save(merged.global.dataset1, file= "merged.global.dataset.1.Rdata")


                                                   ##############################
                                                     ## – Running GLM models -- ##
                                                   ##############################

## -- Long-lat projection -- ##

load(".../merged.global.dataset.1.Rdata")
# If species occupies more than 10% of the cell it was assigned as "present", if not, as 0
merged.global.dataset1[,c(10:21)] <- ifelse(merged.global.dataset1[,c(10:21)] >= 10 ,1 , 0)
merged.global.dataset1[,c(10:21)] <- ifelse(is.na(merged.global.dataset1[,c(10:21)]),0 , 1) # Turn NA to 0 to have absences
merged.global.dataset1 <- merged.global.dataset1[merged.global.dataset1$Landcover>0,]

# Variable transformations

merged.global.dataset1$bio15 <- sqrt(merged.global.dataset1$bio15+2)
merged.global.dataset1$bio12 <- sqrt(merged.global.dataset1$bio12+1)
merged.global.dataset1$bio2 <- merged.global.dataset1$bio2/10
merged.global.dataset1$bio1 <- merged.global.dataset1$bio1/10

variables <- paste("bio1 + I(bio1^2) +
                  bio2 + I(bio2^2) +
                  bio12 + I(bio12^2) +
                  bio15 + I(bio15^2)")

# As is model, no corrections
l_models <- list()

for (i in names(merged.global.dataset1)[10:21]){
  form <- formula(paste(i, "~", variables))
  l_models[[i]] <- glm(form, data=merged.global.dataset1, family='binomial')
}

## weighted by landcover
ll_models <- list()
for (i in names(merged.global.dataset1)[10:21]){
  form <- formula(paste(i, "~", variables))
  ll_models[[i]] <- glm(form, data=merged.global.dataset1,family='binomial',
                        weights= merged.global.dataset1$Landcover)
}
## weighted by cell area (as % of max area, to decrease deviance factors)
la_models <- list()

for (i in names(merged.global.dataset1)[10:21]){
  form <- formula(paste(i, "~", variables))
  la_models[[i]] <- glm(form, data=merged.global.dataset1,family='binomial',
                        weights= cell_area/max(cell_area, na.rm=T))
}


## weighted by cell area AND landcover
lla_models <- list()

for (i in names(merged.global.dataset1)[10:21]){
  form <- formula(paste(i, "~", variables))
  lla_models[[i]] <- glm(form, data=merged.global.dataset1,family='binomial',
                         weights= cell_area/max(cell_area, na.rm=T)*Landcover)
}
```

```
## -- Mollweide models -- ##

load(".../merged.global.dataset.100.Rdata")
# If species occupies more than 10% of the cell it was assigned as "present", if not, as 0
merged.global.dataset100[,c(9:20)] <- ifelse(merged.global.dataset100[,c(9:20)] >= 10 ,1 , 0)
merged.global.dataset100[,c(9:20)] <- ifelse(is.na(merged.global.dataset100[,c(9:20)]),0 , 1) # Turn NA to 0 to have absences
merged.global.dataset100 <- merged.global.dataset100[merged.global.dataset100$Landcover>0,]
# Variable transformations
merged.global.dataset100$bio15 <- sqrt(merged.global.dataset100$bio15+2)
merged.global.dataset100$bio12 <- sqrt(merged.global.dataset100$bio12+1)
merged.global.dataset100$bio2 <- merged.global.dataset100$bio2/10
merged.global.dataset100$bio1 <- merged.global.dataset100$bio1/10

#### Run Mollweide Models

# Uncorrected for landcover
m_models <- list()

for (i in names(merged.global.dataset100)[9:20]){
  form <- formula(paste(i, "~", variables))
  m_models[[i]] <- glm(form, data=merged.global.dataset100, family='binomial')
}


# Weighted by landcover = the reference model
ml_models <- list()

for (i in names(merged.global.dataset100)[9:20]){
  form <- formula(paste(i, "~", variables))
  ml_models[[i]] <- glm(form, data=merged.global.dataset100, family='binomial',
                      weights= merged.global.dataset100$Landcover)
}

list=ls() # Save them all together as one object
save(list=ls(),file="GLM_models.Rdata")

####################################
## -- Model prediction plots -- ##
####################################

# Long-lat dataset
load("/merged.global.dataset.1.Rdata")
merged.global.dataset1[,c(10:21)] <- ifelse(merged.global.dataset1[,c(10:21)] >= 10 ,1 , 0)
merged.global.dataset1[,c(10:21)] <- ifelse(is.na(merged.global.dataset1[,c(10:21)]),0 , 1) # Turn NA to 0 to have absences
merged.global.dataset1 <- merged.global.dataset1[merged.global.dataset1$Landcover>0,]
untransformedData1 <- merged.global.dataset1 # For plotting on original scale
merged.global.dataset1$bio15 <- sqrt(merged.global.dataset1$bio15+2)
merged.global.dataset1$bio12 <- sqrt(merged.global.dataset1$bio12+1)
merged.global.dataset1$bio2 <- merged.global.dataset1$bio2/10
merged.global.dataset1$bio1 <- merged.global.dataset1$bio1/10

# Mollweide dataset
load("/merged.global.dataset.100.Rdata")
merged.global.dataset100[,c(9:20)] <- ifelse(merged.global.dataset100[,c(9:20)] >= 10 ,1 , 0)
merged.global.dataset100[,c(9:20)] <- ifelse(is.na(merged.global.dataset100[,c(9:20)]),0 , 1) # Turn NA to 0 to have absences
merged.global.dataset100 <- merged.global.dataset100[merged.global.dataset100$Landcover>0,]
untransformedData100 <- merged.global.dataset100 # For plotting on original scale
# Variable transformations
merged.global.dataset100$bio15 <- sqrt(merged.global.dataset100$bio15+2)
merged.global.dataset100$bio12 <- sqrt(merged.global.dataset100$bio12+1)
merged.global.dataset100$bio2 <- merged.global.dataset100$bio2/10
merged.global.dataset100$bio1 <- merged.global.dataset100$bio1/10

# set up new temperature data for plotting:
new.temp.df <- cbind.data.frame(bio1=seq(-25, 30, len=300), t(apply(merged.global.dataset1[,5:7], 2, median,na.rm=T)))
new.temp.df.m <- cbind.data.frame(bio1=seq(-25, 30, len=300), t(apply(merged.global.dataset100[,5:7], 2, median,na.rm=T)))


#### reindeer and elk show strongest bias #### 10 and 11
i<-11
# Temperature
preds.Lfit <- predict(l_models[[i]], newdata=new.temp.df, se.fit=T)
preds.LLfit <- predict(ll_models[[i]], newdata=new.temp.df, se.fit=T)
preds.LAfit <- predict(la_models[[i]], newdata=new.temp.df, se.fit=T)
preds.LLAfit <- predict(lla_models[[i]], newdata=new.temp.df, se.fit=T)
preds.Mfit <- predict(m_models[[i]], newdata=new.temp.df.m, se.fit=T)
preds.MLfit <- predict(ml_models[[i]], newdata=new.temp.df.m, se.fit=T)
```

```
plot(Rangifer_tarandus ~ bio1, data=merged.global.dataset1, type="n", ylab="occurrence probability", cex.lab=1.75,
     xlab="mean annual temperature [°C]", axes=F)
axis(1, cex.axis=1.25, labels=seq(-30, 30, by=1), at=seq(-30, 30, by=1))
axis(2, las=1, cex.axis=1.25)
box()

rug(merged.global.dataset1$bio1[merged.global.dataset1$Rangifer_tarandus==0], side=1, col=rgb(0.1,.1,.1,.1))
rug(merged.global.dataset1$bio1[merged.global.dataset1$Rangifer_tarandus==1], side=3, col=rgb(0.1,.1,.1,.1))

# Plot L predictions
polygon(c(new.temp.df$bio1, rev(new.temp.df$bio1)), plogis(c(preds.Lfit$fit+2*preds.Lfit$se.fit,
                                                  rev(preds.Lfit$fit-2*preds.Lfit$se.fit))), col=rgb(.7,.7,.7,.15), border=NA)
lines(new.temp.df$bio1, plogis(preds.Lfit$fit), lwd=3, col="grey90")

# Plot LLA predictions
polygon(c(new.temp.df$bio1, rev(new.temp.df$bio1)), plogis(c(preds.LLAfit$fit+2*preds.LLAfit$se.fit,
                                                  rev(preds.LLAfit$fit-2*preds.LLAfit$se.fit))), col=rgb(.5,.5,.5,.15), border=NA)
lines(new.temp.df$bio1, plogis(preds.Lfit$fit), lwd=3, col="grey70")


# plot M-fit:

polygon(c(new.temp.df.m$bio1, rev(new.temp.df.m$bio1)), plogis(c(preds.Mfit$fit+2*preds.Mfit$se.fit,
                                                  rev(preds.Mfit$fit-2*preds.Mfit$se.fit))), col=rgb(.1,.1,.1,.15), border=NA)
lines(new.temp.df.m$bio1, plogis(preds.Mfit$fit), lwd=3, col="grey30")

# plot ML-fit:
polygon(c(new.temp.df.m$bio1, rev(new.temp.df.m$bio1)), plogis(c(preds.MLfit$fit+2*preds.MLfit$se.fit,
                                                  rev(preds.MLfit$fit-2*preds.MLfit$se.fit))), col=rgb(.1,.1,.1,.15), border=NA)
lines(new.temp.df.m$bio1, plogis(preds.MLfit$fit), lwd=3, col="black")


legend("topright", legend="Reindeer", bty="n", cex=2)
legend("right", legend=c("L", "LLA", "M", "ML"), lwd=3, col=c("grey90", "grey70", "grey30", "black"), bty="n", cex=1.5)


## -- Precipitation -- ##

new.precip.df <- cbind.data.frame(bio12=seq(1, 4000, len=2000), t(apply(merged.global.dataset1[,c(4:5,7)], 2, median,na.rm=T)))
new.precip.df.m <- cbind.data.frame(bio12=seq(1, 4000, len=2000), t(apply(merged.global.dataset100[,c(4:5,7)], 2, median,na.rm=T)))
i<-11
preds.Lfit <- predict(l_models[[i]], newdata=new.precip.df, se.fit=T)
preds.LLfit <- predict(ll_models[[i]], newdata=new.precip.df, se.fit=T)
preds.LAfit <- predict(la_models[[i]], newdata=new.precip.df, se.fit=T)
preds.LLAfit <- predict(lla_models[[i]], newdata=new.precip.df, se.fit=T)
preds.Mfit <- predict(m_models[[i]], newdata=new.precip.df.m, se.fit=T)
preds.MLfit <- predict(ml_models[[i]], newdata=new.precip.df.m, se.fit=T)

backtransfBio12 <- (new.precip.df$bio12^2)-1
quantile(untransformedData1$bio12, 0.99, na.rm=T)
# use untransformed dataset for presence absence plotting
plot(get(names(l_models)[i]) ~ bio12, data=untransformedData1, type="n", xlim=(c(0,1631)), ylab="occurrence probability", cex.lab=1.75,
     xlab="annual precipitation [mm]", axes=F)
axis(1, cex.axis=1.25, labels=seq(1, 1631, by=100), at=seq(1, 1631, by=100))
axis(2, las=1, cex.axis=1.25)
box()

rug(untransformedData1$bio12[untransformedData1$Rangifer_tarandus==0], side=1, col=rgb(0.1,.1,.1,.1))
rug(untransformedData1$bio12[untransformedData1$Rangifer_tarandus==1], side=3, col=rgb(0.1,.1,.1,.1))

# Plot L predictions
polygon(c(backtransfBio12, rev(backtransfBio12)), plogis(c(preds.Lfit$fit+2*preds.Lfit$se.fit,
                                                 rev(preds.Lfit$fit-2*preds.Lfit$se.fit))), col=rgb(.7,.7,.7,.15), border=NA)
lines(backtransfBio12, plogis(preds.Lfit$fit), lwd=3, col="grey90")

# Plot LLA predictions
polygon(c(backtransfBio12, rev(backtransfBio12)), plogis(c(preds.LLAfit$fit+2*preds.LLAfit$se.fit,
                                                 rev(preds.LLAfit$fit-2*preds.LLAfit$se.fit))), col=rgb(.5,.5,.5,.15), border=NA)
lines(backtransfBio12, plogis(preds.Lfit$fit), lwd=3, col="grey70")

# plot M-fit:
backtransfBio12m <- (new.precip.df.m$bio12^2)-1
polygon(c(backtransfBio12, rev(backtransfBio12)), plogis(c(preds.Mfit$fit+2*preds.Mfit$se.fit,
                                                 rev(preds.Mfit$fit-2*preds.Mfit$se.fit))), col=rgb(.1,.1,.1,.15), border=NA)
lines(backtransfBio12, plogis(preds.Mfit$fit), lwd=3, col="grey30")

# plot ML-fit:
polygon(c(backtransfBio12, rev(backtransfBio12)), plogis(c(preds.MLfit$fit+2*preds.MLfit$se.fit,
                                                 rev(preds.MLfit$fit-2*preds.MLfit$se.fit))), col=rgb(.1,.1,.1,.15), border=NA)
```

```
lines(backtransfBio12, plogis(preds.MLfit$fit), lwd=3, col="black")

legend("topright", legend="Reindeer", bty="n", cex=2)
legend(0,1.02, legend=c("L", "LLA", "M", "ML"), lwd=3, col=c("grey90", "grey70", "grey30", "black"), bty="n", cex=1.5)



## -- Predicted and original range size – ##


# Datasets are are already loaded from previous point

predRangeSize <- as.data.frame(matrix(nrow=12,ncol=9))
colnames(predRangeSize) <- c("species", "L_originalSize", "M_originalSize", "l","la","ll","lla","m","ml")
predRangeSize$species <- names(l_models)

# Calculate the original and predicted range size
for (i in 1:12){
  predRangeSize[i, "L_originalSize"] <- sum(merged.global.dataset1[,names(l_models)[i]] * merged.global.dataset1$cell_area, na.rm=T)
  predRangeSize[i, "M_originalSize"] <- sum(merged.global.dataset100[,names(l_models)[i]] * 10000, na.rm=T)
  predRangeSize[i, "l"] <- sum(plogis(predict(l_models[[i]], newdata=merged.global.dataset1)) * merged.global.dataset1$cell_area, na.rm=T)
  predRangeSize[i, "ll"] <- sum(plogis(predict(ll_models[[i]], newdata=merged.global.dataset1)) * merged.global.dataset1$cell_area, na.rm=T)
  predRangeSize[i, "la"] <- sum(plogis(predict(la_models[[i]], newdata=merged.global.dataset1)) * merged.global.dataset1$cell_area, na.rm=T)
  predRangeSize[i, "lla"] <- sum(plogis(predict(lla_models[[i]], newdata=merged.global.dataset1)) * merged.global.dataset1$cell_area, na.rm=T)
  predRangeSize[i, "m"] <- sum(plogis(predict(m_models[[i]], newdata=merged.global.dataset100)) * 10000, na.rm=T)
  predRangeSize[i, "ml"] <- sum(plogis(predict(ml_models[[i]], newdata=merged.global.dataset100)) * 10000, na.rm=T)
}


predRangeSize[,2:9] <- predRangeSize[,2:9]/1000000 (expressed as 10^6km)
predRangeSize
boxplot(predRangeSize[2:9],ylab="range size")


                         ##########################
                          ##### Relative bias #####
                         ##########################


# Bias for every parameter estimate, for every species
# Rel bias species A = ||coeff_i_model_i| - |coeff_i_model_ref|| / se(coef_i_model_ref)|
# The smaller the standard error of the reference model, the higher the bias.
# The second formula weights each parameter by it's partial explained deviance in the model
# Important parameters will have a greater contribution to bias estimate.

# Function to calculate relative bias
calcBias <- function(x,y){
  # Relative bias for every parameter of model x, for species i
  paramRelBias <- abs((x$coefficients[-1] - y$coefficients[-1]) / coef(summary(y))[-1,2] )
  # now every parameter in the model x has to be weighted by its deviance (e.g. temp * (deviance temp)/(sum of deviances of all parameters))
  # parameters of less importance will have lower weight in the model
  # the mean bias of all parameters is then calculated for each species
  result <- mean(paramRelBias * anova(x)[-1,2]/sum(anova(x)[-1,2]))
  return(result)
}

relBias <- as.data.frame(matrix(nrow=12,ncol=5),row.names=names(l_models))
colnames(relBias) <- c("l","la","ll","lla","m")

models <- c("l","la","ll","lla","m")
for(i in models){
  relBias[,i] <- mapply(calcBias, get(paste(i,"_models",sep="")), ml_models)
}

summary(relBias)

# -- Extracting parameters for relative bias analyses -- ##

# min, max, mean, median latitude and latitude range of each species

range_lat <- as.data.frame(matrix(nrow=12, ncol=5), row.names=names(merged.global.dataset1)[10:21])
colnames(range_lat) <- c("minLat","maxLat","meanLat","medianLat", "rangeSize")
for(i in 1:12){
  range_lat[i,] <- round(c(min(merged.global.dataset1$y[merged.global.dataset1[rownames(range_lat)[i]]==1],na.rm=T),
                        max(merged.global.dataset1$y[merged.global.dataset1[rownames(range_lat)[i]]==1],na.rm=T),
                        mean(merged.global.dataset1$y[merged.global.dataset1[rownames(range_lat)[i]]==1],na.rm=T),
                        median(merged.global.dataset1$y[merged.global.dataset1[rownames(range_lat)[i]]==1],na.rm=T),
                        sum(merged.global.dataset1[which(merged.global.dataset1[,rownames(range_lat)[i]]==1), "cell_area"])))
}
```

```
## -- Find the R-sq for each model -- ##

load("GLM_models.Rdata")
Rsq <- as.data.frame(matrix(nrow=12, ncol=5), row.names=names(l_models))
colnames(Rsq) <- c("l","la","ll","lla","m")

for(i in colnames(Rsq)){
  Rsq[,i] <- sapply(get(paste(i,"_models",sep="")), function(x) 1-(x$deviance/x$null.deviance))
}
## -- Stacking -- ##

Rsq_stack <- data.frame(stack(Rsq), species=rownames(Rsq))
colnames(Rsq_stack)[1] <- "Rsq"
Rsq_stack$ID <- paste(Rsq_stack$species,Rsq_stack$ind, sep="") # Create and ID
Rsq_stack<- Rsq_stack[,c("ID","Rsq")]

relB <- data.frame(stack(relBias), species = rownames(relBias))
colnames(relB)[1:2] <- c("relBias", "model")
relB$proj <- c(rep("long-lat",48),rep("moll",12)) # add projections
relB$ID <- paste(relB$species, relB$model, sep="") # Create an ID for merging without errors

# Merge them
relBias <- merge(relB, Rsq_stack, by= "ID")
# Add other variables
range_lat$species <- rownames(range_lat)
biasAnalysis <- merge(relBias, range_lat, by="species")
biasAnalysis <- biasAnalysis[order(biasAnalysis$model),]
# Manually add other variables
biasAnalysis$LandcoverCorr <- c(rep(0,24),rep(1,24),rep(0,12))
biasAnalysis$AreaCorr <- c(rep(0,12),rep(1,12),rep(0,12),rep(1,24))
biasAnalysis <- biasAnalysis[order(biasAnalysis$species),]
biasAnalysis$coastal <- c(rep(1,5),rep(0,5),rep(1,10),rep(0,5),rep(1,10),rep(0,5),rep(1,10),rep(0,5),rep(1,5))
biasAnalysis$rangeLat <- biasAnalysis$maxLat - biasAnalysis$minLat

                                 ################################
                                     ## ----- Bias analysis ----- ##
                                 ################################

## -- bias among models -- ##

fm <- lm(log(relBias+0.01) ~ model, data=biasAnalysis)
anova(fm)
TukeyHSD(aov(fm))


## -- Mixed effect models -- ##

require(effects)
require(nlme)
#manually simplified:
fm <- lme(log(relBias+0.01) ~ maxLat +log(rangeSize) +LandcoverCorr * coastal   + proj + AreaCorr, random=~1|species, data=biasAnalysis)
anova(fm)
summary(fm)

boxplot(relBias ~ model,data=biasAnalysis, whisklty=1, col="grey50", las=1)

# Find the mean relative bias for each projection
aggregate(.~model, FUN=mean, data=biasAnalysis[, 3:4])
aggregate(.~species, FUN=mean, data=biasAnalysis[, c(1,3)]) # elk and reindeer

## -- figure: individual biases ~ distribution size -- ##
attach(biasAnalysis)
rangeSize <- rangeSize/1000000
col <- sapply(model, function(x) switch(x, "l"="white", "ll"="grey90", "la"="grey50", "lla"="grey20", "m"="grey70"))
sym <- sapply(model, function(x) switch(x, "l"=21, "ll"=21, "la"=21, "lla"=21, "m"=22))
par(mar=c(5,5,1,1))
plot(biasAnalysis$relBias ~ rangeSize, las=1, bg=col, pch=sym, cex=2, log="x", type="n", xlab=expression(paste("range size [", 10^6, " ", km^2,
"]")),
     ylab="relative bias", cex.lab=1.6, cex.axis=1.25,mgp=c(3.5,0.5,0))
abline(h=0, lwd=2, col="grey")
points(biasAnalysis$relBias ~ rangeSize, bg=col, pch=sym, cex=2)
legend("topleft", bty="n", pch=c(21, 21, 21, 21, 22), pt.bg=c("white", "grey90", "grey50", "grey20", "grey70"), legend=c("L", "LL", "LA", "LLA",
"M"), cex=1.5)
text(tapply(rangeSize, species, max), tapply(biasAnalysis$relBias, species, max)+0.01+c(0,0,-0.12,0,0,0,0,-0.1,0,0,0,-0.08),
     c("Eurasian elk", "Bactrian camel *",  "Siberian roe deer", "Red deer",
       "Alpine musk deer *", "Mule deer", "Muskox", "Wild sheep *", "Bighorn sheep", "Thinhorn sheep", "Mongolian gazelle *","Reindeer"),
```

```
    srt=90, pos=4, offset=-.1, font=3, cex=0.9)

## -- figure: bias for each model -- ##
biasAnalysis$model <- factor(biasAnalysis$model, levels=c("l", "ll", "la", "lla", "m"))
plot(biasAnalysis$model,biasAnalysis$relBias,at=c(1,2,3,4,5),type="n", ylab="relative bias [SD multiples]", cex.lab=2,whisklty=1,col=c("white", "grey90", "grey70", "grey50", "grey45"),axes=F,xlab="model variants", cex.lab=1.4)
abline(h=0, col="grey", lwd=2)
axis(side=2, las=1)
axis(1,at=1:5, labels=c("L", "LL", "LA", "LLA", "M"),tick=F)
box()



## -- Additional analyses: model differences – ##

## -- Coefficients SE among models -- ##

# Relative bias calculation is influenced by the coeffient standard error of in the reference model (ML).
# We test here whether standard errors differ among coefficients in different model variants.

errorEst <- as.data.frame(matrix(nrow=72,ncol=10))
colnames(errorEst) <- c("model", "species","bio1","bio1sq","bio2","bio2sq","bio12","bio12sq","bio15","bio15sq")
errorEst$species <- rep(names(l_models), 6)
errorEst$model <- rep(c("l","la","ll","lla","m","ml"),each=12)
errorEst$proj <- c(rep("variant",60),rep("ref",12))

for(i in unique(errorEst$model)){
  errorEst[which(errorEst$model==i),3:10] <-t(sapply(get(paste(i,"_models",sep="")), function(x) coef(summary(x))[-1,2]))
}

# linear model coefficientSE ~ model_variant
apply(errorEst[,3:10], 2, function(x) anova(lm(x~model,data = errorEst)))


## -- Predictors partial explained deviances -- ##

paramWeights <- as.data.frame(matrix(nrow=72,ncol=11))
colnames(paramWeights) <- c("model","areaWeight", "species","bio1","bio1sq","bio2","bio2sq","bio12","bio12sq","bio15","bio15sq")
paramWeights$species <- rep(names(l_models), 6)
paramWeights$model <- rep(c("l","la","ll","lla","m","ml"),each=12)
paramWeights$areaWeight <- c(rep(0,12),rep(1,12),rep(0,12),rep(1,36))
paramWeights$proj <- c(rep("l",48),rep("m",24))

for(i in unique(paramWeights$model)){
  paramWeights[which(paramWeights$model==i),4:11] <-t(sapply(get(paste(i,"_models",sep="")), function(x) anova(x)[-1,2]/sum(anova(x)[-1,2])))
}

attach(paramWeights)
library(nlme)

anova(flm1 <- lme(bio1 + bio1sq ~ areaWeight, random=~1|species, data=paramWeights))
anova(lme(bio2 +bio2sq ~ areaWeight, random=~1|species, data=paramWeights))
anova(lme(bio12 +bio12sq~ areaWeight, random=~1|species, data=paramWeights))
anova(lme(bio15+bio15sq ~ areaWeight, random=~1|species, data=paramWeights))
# bio2, bio12 and bio15 at 0.05 level differ between with/our areaWeight,
# bio2 differ among projection types (M/L) (bio12 and bio15 marginally 0.06-0.07)
# bio2 differ among models


## -- Predictors absolute estimates among models -- ##

paramEst <- as.data.frame(matrix(nrow=72,ncol=10))
colnames(paramEst) <- c("model", "species","bio1","bio1sq","bio2","bio2sq","bio12","bio12sq","bio15","bio15sq")
paramEst$species <- rep(names(l_models), 6)
paramEst$model <- rep(c("l","la","ll","lla","m","ml"),each=12)
paramEst$proj <- c(rep("l",48),rep("m",24))

for(i in unique(paramEst$model)){
  paramEst[which(paramEst$model==i),3:10] <-t(sapply(get(paste(i,"_models",sep="")), function(x) abs(x$coefficients[-1])))
}

summary(flm1 <- lme(bio1 ~ proj, random=~1|species, data=paramEst))
summary(lme(bio1sq ~ proj, random=~1|species, data=paramEst))
summary(lme(bio2 ~ proj, random=~1|species, data=paramEst))
summary(lme(bio2sq ~ proj, random=~1|species, data=paramEst))
summary(lme(bio12 ~ proj, random=~1|species, data=paramEst))
summary(lme(bio12sq ~ proj, random=~1|species, data=paramEst))
summary(lme(bio15 ~ proj, random=~1|species, data=paramEst))
summary(lme(bio15sq ~ proj, random=~1|species, data=paramEst))
```

```
# so bio1 and bio1sq  differ between projections, bio15 at 0.05 level
### additional test: if regression dillution occurrs more in non-area-weighted models among lon-lat versions -- NO, it does not

predict(flm1, newdata=data.frame("proj"=c("l","m")), level=0) # difference between with/out area-correction

library(AICcmodavg)
preds <- predictSE.lme(flm1, newdata=data.frame("proj"=c("1","m"))) # population level
barplot(preds$fit, ylim=c(0, .5), las=1, names.arg = c("l", "m"), ylab="parameter estimate for bio1", cex.lab=2)
arrows(0.7, preds$fit[1]-preds$se.fit[1], 0.7, preds$fit[1]+preds$se.fit[1], code=3, angle=90, lwd=2)
arrows(1.9, preds$fit[2]-preds$se.fit[2], 1.9, preds$fit[2]+preds$se.fit[2], code=3, angle=90, lwd=2)
```
