# Supplementary material for: Squares of different sizes: effect of geographical projection on model parameter estimates in species distribution modeling
Source: Ecol Evol. 2015 Dec 17;6(1):202–11. doi: 10.1002/ece3.1838 (PMC4716504; doi:10.1002/ece3.1838)
Supplement: Supplementary file 2 [file ECE3-6-202-s002.odt]

# Contents

# 1. extracting ungulates to 1x1 degree and 100x100 km grid

# 2. Extracting environmental variables

# 3. Running GLM species distribution models for the two projection types

# 4. Calculating relative bias in parameter estimates among model variants

library(raster)

library(rgdal)

library(maptools)

library(lattice)

# Load IUCN shape file

mams <- readShapeSpatial("/.../TERRESTRIAL_MAMMALS") # unprojected, 

proj4string(mams)<- CRS("+proj=longlat +datum=WGS84") # the datum (wgs84) is written in metadata and added manually

# Function to extract species

extract.species <- function(mams, species){

  # SPDF is the spatialPolygonsDataFrame object

  # species.name of the species to be extracted

  # author: Carsten F. Dormann

  if ("binomial" %in% names(mams@data))  ind <- which(mams@data$binomial == species)

  selected.species <- mams

  selected.species@data <- mams@data[ind,]

  selected.species@polygons <- mams@polygons[ind]

  selected.species@plotOrder <- seq_along(ind)  

  return(selected.species)

}

# Ungulates to extract

species <- c("Moschus chrysogaster", "Odocoileus hemionus", "Capreolus pygargus", "Procapra gutturosa", "Camelus ferus", "Ovis ammon", "Ovis dalli", "Ovis canadensis", "Cervus elaphus", "Alces alces", "Rangifer tarandus", "Ovibos moschatus")

## -- Extract species to 1 degree grid -- ##

ll <- raster(mams) # Crate an empty raster with 1 degree resolution

res(ll) <-1 # set the resolution to 1 degree

save(ll, file="empty_raster_1deg_iucn_extent.Rdata")

raster.ungulates <- rasterize(extract.species(mams, species[1]), ll)

for (i in 2:length(species)){

  raster.ungulates2 <- rasterize(extract.species(mams, species[i]), ll)

  raster.ungulates <- addLayer(raster.ungulates, raster.ungulates2)

  print(paste("Finished the ", i, "th species: ", species[i], sep=""))

}

species <- gsub(" ","_",species)

names(raster.ungulates) <- species

## -- Eliminating any non-Holarctic ranges -- #

# The extent of the raster remain the same, but any values outside the holarctic polygon will be turned to NA

# source http://www.arcgis.com/home/item.html?id=27eef65481234036bdf55a78150e1f9d

setwd(".../gen_bio_realm")

bio_realms <- readShapeSpatial("gen_bio_realm_2004.shp") # GEt the shape of Holarctic

holarctic <- SpatialPolygons(bio_realms@polygons[c(1,2,3,4)])

proj4string(holarctic)<- CRS("+proj=longlat +datum=WGS84") # set the coordinate system

# from the suggestion (https://stat.ethz.ch/pipermail/r-sig-geo/2012-June/015260.html)

crop  <- setValues(raster.ungulates, NA) # Create a dummy NA raster with a spatial extension equal to the cropped raster

myshp.r <- rasterize(holarctic, crop) #  Rasterize the catchment boundaries, with NA outside the catchment boundaries

ungulates.hol <- mask(x=raster.ungulates, mask=myshp.r)

plot(ungulates.hol$Rangifer_tarandus)

plot(holarctic, add=T)

# extract the coordinates and save in a dataframe format

coord <- as.data.frame(xyFromCell(ungulates.hol, 1:ncell(ungulates.hol))) # extract the coordinates.

ungulates_cover_1deg <- cbind.data.frame(coord, ungulates = values(ungulates.hol)) # bind them to the occurrence data

colnames(ungulates_cover_1deg) <- gsub("ungulates.","",colnames(ungulates_cover_1deg))

ungulates_cover_1deg$ID <- paste(ungulates_cover_1deg$x, ungulates_cover_1deg$y, sep="+")

save(ungulates_cover_1deg, file="ungulates_cover_1degree.Rdata")

## -- Extracting ungulates to 100 x 100 Mollweide -- ##

# The same as before, just with different projection

# Transform IUCN data to mollweide equal area projection

mams <- spTransform(mams, CRS('+proj=moll')) 

# Create an empty raster in equal area projection with 100 x 100 km reslution and the same extent as mammals range polygons

moll <- raster(mams)

res(moll) <- 100000 # set the resolution

save(moll, file = "empty_raster_100km_iucn_extent.Rdata")

species <- gsub("_"," ",species)

raster.ungulates <- rasterize(extract.species(mams, species[1]), moll)

for (i in 2:length(species)){

  raster.ungulates2 <- rasterize(extract.species(mams, species[i]), moll)

  raster.ungulates <- addLayer(raster.ungulates, raster.ungulates2)

  print(paste("Finished the ", i, "th species: ", species[i], sep=""))

}

species <- gsub(" ","_",species)

names(raster.ungulates) <- species

# Remove non-holarctic ranges

holarctic_moll <- spTransform(holarctic, "+proj=moll") # set the Mollweide projection to holarctic

crop  <- setValues(raster.ungulates, NA)

myshp.r <- rasterize(holarctic_moll, crop)

ungulates.hol <- mask(x=raster.ungulates, mask=myshp.r)

plot(ungulates.hol$Rangifer_tarandus)

plot(holarctic_moll, add=T)

# extract coordinates and save in a dataframe format.

coord <- as.data.frame(xyFromCell(ungulates.hol, 1:ncell(ungulates.hol)))

ungulates_cover_Moll_100 <- cbind.data.frame(coord, ungulates =values(ungulates.hol))

colnames(ungulates_cover_Moll_100) <- gsub("ungulates.","",colnames(ungulates_cover_Moll_100))

ungulates_cover_Moll_100$ID <- paste(ungulates_cover_Moll_100$x, ungulates_cover_Moll_100$y, sep="+")

levelplot(Rangifer_tarandus ~x+y,data=ungulates_cover_Moll_100) # test plot

save(ungulates_cover_Moll_100, file="ungulates_cover_Mollweide_100.Rdata")

#####################################################

### --  Extracting environmental data -- ###

#####################################################

## -- Bioclim 1 deg -- ##

# Load Biolicm data

setwd(".../Raw_data")

bioclim.data <- getData('worldclim', var='bio', res=10, download=T) # 27.02.2015

# It is a rasterstack, CRS defined

# Aggregate to coarster resolution (1 deg). Water bodies have NA values so averaging will give correct outcome

bioclim <- projectRaster(from = bioclim.data, to=ll) 

area <- area(ll) # Get the area of each cell in long-lat projection

names(area) <- "cell_area"

bioclim <- addLayer(bioclim, area)

# cut out non-holarctic areas

crop  <- setValues(bioclim, NA)

myshp.r <- rasterize(holarctic, crop)

bioclim.hol <- mask(x=bioclim, mask=myshp.r)

plot(bioclim.hol$cell_area)

plot(holarctic, add=T)

# Extracting the coordinates and saving in a dataframe.

coord <- as.data.frame(xyFromCell(bioclim.hol, 1:ncell(bioclim.hol)))

bioclim_cover <- cbind.data.frame(coord, bioclim =values(bioclim.hol))

colnames(bioclim_cover) <- gsub("bioclim.","",colnames(bioclim_cover))

bioclim_cover$ID <- paste(bioclim_cover$x, bioclim_cover$y, sep="+")

levelplot(bio1 ~x+y,data=bioclim_cover) # check how it looks

save(bioclim_cover, file="bioclim_cover.grid_1deg_holarctic.Rdata")

## -- Bioclim 100x100 Mollweide -- ##

# Change projection

bioclim <- projectRaster(from = bioclim.data, to=moll)

# Cut to holarctic

crop  <- setValues(bioclim, NA)

myshp.r <- rasterize(holarctic_moll, crop)

bioclim.hol <- mask(x=bioclim, mask=myshp.r)

plot(bioclim.hol$bio1)

plot(holarctic_moll, add=T)

# Extracting the coordinates and saving in a dataframe.

coord <- as.data.frame(xyFromCell(bioclim.hol, 1:ncell(bioclim.hol)))  # this extracts the coordinates.

bioclim_cover <- cbind.data.frame(coord, bioclim =values(bioclim.hol))

colnames(bioclim_cover) <- gsub("bioclim.","",colnames(bioclim_cover))

bioclim_cover$ID <- paste(bioclim_cover$x, bioclim_cover$y, sep="+")

save(bioclim_cover, file="bioclim_cover.grid_Moll100_holarctic.Rdata")

## -- Global land cover 1 degree -- ##

# Get GLC data

setwd(".../GLC2000_Tiff")

glc<-raster("glc2000_v1_1.tif")

load("~/new_IUCN/New_globaldataset/projections/empty_raster_1deg_iucn_extent.Rdata") # Load the empty raster

glcTerr <- glc ==20 # Terrestrial cover, original data is binary (0,1)

# To aggregate Terrestrial cover to coarser resolution, cells covered by water SHOULD NOT be turned to NA

# We need to know the percentage of land in a cell

glcTerrCoarse <- aggregate(glcTerr, fact=16, fun=mean, expand=TRUE, na.rm=TRUE)

values(glcTerrCoarse) <- (1-values(glcTerrCoarse))/1 # swap water and land values.

plot(glcTerrCoarse,xlim=c(-30,30),ylim=c(-20,20)) # perfect

projection(glcTerrCoarse) <- CRS("+proj=longlat +ellps=WGS84 +datum=WGS84 +no_defs +towgs84=0,0,0") # set the coordinate system

glcTerr_grid1 <- projectRaster(glcTerrCoarse, ll) 

plot(glcTerr_grid1,xlim=c(-30,30),ylim=c(-20,20))

save(glcTerr_grid1, file= "landcover_grid1.Rdata")

# Cut to holarctic area

crop  <- setValues(glcTerr_grid1, NA) # Dummy raster with a spatial extension equal to the cropped raster,but full of NA values

myshp.r <- rasterize(holarctic, crop) #  Rasterize the catchment boundaries, with NA outside the catchment boundaries

glcTerr_grid1.hol <- mask(x=glcTerr_grid1, mask=myshp.r)

plot(glcTerr_grid1.hol$layer) # check how it looks

# Extracting coordinates and saving in data frame.

coord <- as.data.frame(xyFromCell(glcTerr_grid1.hol, 1:ncell(glcTerr_grid1.hol)))  

Terr_cover_grid1 <- cbind.data.frame(coord, Landcover =values(glcTerr_grid1.hol)) 

Terr_cover_grid1$ID <- paste(Terr_cover_grid1$x, Terr_cover_grid1$y, sep="+")

save(Terr_cover_grid1, file=".../terrestrialCoverFromGLC20_grid1deg_holarctic.Rdata")

## -- Terrestrial cover 100 x 100 – ##

glcTerr_grid100 <- projectRaster(glcTerrCoarse, moll) 

plot(glcTerr_grid100,xlim=c(-3000000,3000000),ylim=c(-2000000,2000000)) # Looks good!

save(glcTerr_grid100, file= "landcover_grid100.Rdata")

crop  <- setValues(glcTerr_grid100, NA) # Dummy raster with a spatial extension equal to the cropped raster,but full of NA values

myshp.r <- rasterize(holarctic_moll, crop) #  Rasterize the catchment boundaries, with NA outside the catchment boundaries

# Assigning NA values to all  raster cells outside holarctic shapefile boundaries

glcTerr_grid100.hol <- mask(x=glcTerr_grid100, mask=myshp.r)

plot(glcTerr_grid100.hol$layer) # check how it looks

# Extracting coordinates and saving in data frame.

coord <- as.data.frame(xyFromCell(glcTerr_grid100.hol, 1:ncell(glcTerr_grid100.hol)))  

Terr_cover_grid100 <- cbind.data.frame(coord, Landcover =values(glcTerr_grid100.hol)) 

Terr_cover_grid100$ID <- paste(Terr_cover_grid100$x, Terr_cover_grid100$y, sep="+")

save(Terr_cover_grid100, file=".../terrestrialCoverFromGLC20_grid100_holarctic.Rdata")

levelplot(Landcover~ x + y, data = Terr_cover_grid100)

###   Merging environmental data ###

load("~/new_IUCN/New_globaldataset/projections/bioclim_cover.grid_Moll100_holarctic.Rdata")

load("~/new_IUCN/New_globaldataset/projections/ungulates_cover_Mollweide_100.Rdata")

load("~/new_IUCN/New_globaldataset/projections/terrestrialCoverFromGLC20_grid100_holarctic.Rdata")

list.of.data.frames100 = list(bioclim_cover[,c("x","y","bio1", "bio2", "bio12", "bio15", "ID")], 

                              Terr_cover_grid100[,c("Landcover","ID")],

                              ungulates_cover_Moll_100[,3:15]) 

merged.global.dataset100 = Reduce(function(...) merge(..., all=T, by="ID"), list.of.data.frames100)

merged.global.dataset100[which(merged.global.dataset100$Landcover==0),c(4:20)] <- NA

save(merged.global.dataset100, file= "merged.global.dataset.100.Rdata")

######################################

load("~/new_IUCN/New_globaldataset/projections/bioclim_cover.grid_1deg_holarctic.Rdata")

load("~/new_IUCN/New_globaldataset/projections/ungulates_cover_1degree.Rdata")

load("~/new_IUCN/New_globaldataset/projections/terrestrialCoverFromGLC20_grid1deg_holarctic.Rdata")

list.of.data.frames1 = list(bioclim_cover[,c("x", "y", "bio1", "bio2", "bio12", "bio15", "cell_area", "ID")], 

                            Terr_cover_grid1[,c("Landcover","ID")],

                            ungulates_cover_1deg[,3:15])  

merged.global.dataset1 = Reduce(function(...) merge(..., all=T, by="ID"), list.of.data.frames1)

merged.global.dataset1[which(merged.global.dataset1$Landcover==0),c(4:8,9:21)] <- NA

save(merged.global.dataset1, file= "merged.global.dataset.1.Rdata")

#############################

## – Running GLM models -- ##

#############################

## -- Long-lat projection -- ##

load(".../merged.global.dataset.1.Rdata")

# If species occupies more than 10% of the cell it was assigned as "present", if not, as 0

merged.global.dataset1[,c(10:21)] <- ifelse(merged.global.dataset1[,c(10:21)] >= 10 ,1 , 0)

merged.global.dataset1[,c(10:21)] <- ifelse(is.na(merged.global.dataset1[,c(10:21)]),0 , 1) # Turn NA to 0 to have absences

merged.global.dataset1 <- merged.global.dataset1[merged.global.dataset1$Landcover>0,]

# Variable transformations

merged.global.dataset1$bio15 <- sqrt(merged.global.dataset1$bio15+2)

merged.global.dataset1$bio12 <- sqrt(merged.global.dataset1$bio12+1)

merged.global.dataset1$bio2 <- merged.global.dataset1$bio2/10

merged.global.dataset1$bio1 <- merged.global.dataset1$bio1/10

variables <- paste("bio1 + I(bio1^2) + 

                   bio2 + I(bio2^2) +

                   bio12 + I(bio12^2) +

                   bio15 + I(bio15^2)")

# As is model, no corrections

l_models <- list()

for (i in names(merged.global.dataset1)[10:21]){

  form <- formula(paste(i, "~", variables))

  l_models[[i]] <- glm(form, data=merged.global.dataset1, family='binomial') 

}

## weighted by landcover

ll_models <- list()

for (i in names(merged.global.dataset1)[10:21]){

  form <- formula(paste(i, "~", variables))

  ll_models[[i]] <- glm(form, data=merged.global.dataset1,family='binomial',

                        weights= merged.global.dataset1$Landcover) 

}

## weighted by cell area (as % of max area, to decrease deviance factors)

la_models <- list()

for (i in names(merged.global.dataset1)[10:21]){

  form <- formula(paste(i, "~", variables))

  la_models[[i]] <- glm(form, data=merged.global.dataset1,family='binomial',

                        weights= cell_area/max(cell_area, na.rm=T)) 

}

## weighted by cell area AND landcover

lla_models <- list()

for (i in names(merged.global.dataset1)[10:21]){

  form <- formula(paste(i, "~", variables))

  lla_models[[i]] <- glm(form, data=merged.global.dataset1,family='binomial',

                         weights= cell_area/max(cell_area, na.rm=T)*Landcover) 

}

## -- Mollweide models -- ##

load(".../merged.global.dataset.100.Rdata")

# If species occupies more than 10% of the cell it was assigned as "present", if not, as 0

merged.global.dataset100[,c(9:20)] <- ifelse(merged.global.dataset100[,c(9:20)] >= 10 ,1 , 0)

merged.global.dataset100[,c(9:20)] <- ifelse(is.na(merged.global.dataset100[,c(9:20)]),0 , 1) # Turn NA to 0 to have absences

merged.global.dataset100 <- merged.global.dataset100[merged.global.dataset100$Landcover>0,]

# Variable transformations

merged.global.dataset100$bio15 <- sqrt(merged.global.dataset100$bio15+2)

merged.global.dataset100$bio12 <- sqrt(merged.global.dataset100$bio12+1)

merged.global.dataset100$bio2 <- merged.global.dataset100$bio2/10

merged.global.dataset100$bio1 <- merged.global.dataset100$bio1/10

#### Run Mollweide Models

# Uncorrected for landcover

m_models <- list()

for (i in names(merged.global.dataset100)[9:20]){

  form <- formula(paste(i, "~", variables))

  m_models[[i]] <- glm(form, data=merged.global.dataset100, family='binomial') 

}

# Weighted by landcover = the reference model

ml_models <- list()

for (i in names(merged.global.dataset100)[9:20]){

  form <- formula(paste(i, "~", variables))

  ml_models[[i]] <- glm(form, data=merged.global.dataset100, family='binomial',

                        weights= merged.global.dataset100$Landcover) 

}

list=ls() # Save them all together as one object

save(list=ls(),file="GLM_models.Rdata")

###################################

## -- Model prediction plots -- ##

###################################

# Long-lat dataset

load("/merged.global.dataset.1.Rdata")

merged.global.dataset1[,c(10:21)] <- ifelse(merged.global.dataset1[,c(10:21)] >= 10 ,1 , 0)

merged.global.dataset1[,c(10:21)] <- ifelse(is.na(merged.global.dataset1[,c(10:21)]),0 , 1) # Turn NA to 0 to have absences

merged.global.dataset1 <- merged.global.dataset1[merged.global.dataset1$Landcover>0,]

untransformedData1 <- merged.global.dataset1 # For plotting on original scale

merged.global.dataset1$bio15 <- sqrt(merged.global.dataset1$bio15+2)

merged.global.dataset1$bio12 <- sqrt(merged.global.dataset1$bio12+1)

merged.global.dataset1$bio2 <- merged.global.dataset1$bio2/10

merged.global.dataset1$bio1 <- merged.global.dataset1$bio1/10

# Mollweide dataset

load("/merged.global.dataset.100.Rdata")

merged.global.dataset100[,c(9:20)] <- ifelse(merged.global.dataset100[,c(9:20)] >= 10 ,1 , 0)

merged.global.dataset100[,c(9:20)] <- ifelse(is.na(merged.global.dataset100[,c(9:20)]),0 , 1) # Turn NA to 0 to have absences

merged.global.dataset100 <- merged.global.dataset100[merged.global.dataset100$Landcover>0,]

untransformedData100 <- merged.global.dataset100 # For plotting on original scale

# Variable transformations

merged.global.dataset100$bio15 <- sqrt(merged.global.dataset100$bio15+2)

merged.global.dataset100$bio12 <- sqrt(merged.global.dataset100$bio12+1)

merged.global.dataset100$bio2 <- merged.global.dataset100$bio2/10

merged.global.dataset100$bio1 <- merged.global.dataset100$bio1/10

# set up new temperature data for plotting:

new.temp.df <- cbind.data.frame(bio1=seq(-25, 30, len=300), t(apply(merged.global.dataset1[,5:7], 2, median,na.rm=T)))

new.temp.df.m <- cbind.data.frame(bio1=seq(-25, 30, len=300), t(apply(merged.global.dataset100[,5:7], 2, median,na.rm=T)))

#### reindeer and elk show strongest bias #### 10 and 11

i<-11

# Temperature

preds.Lfit <- predict(l_models[[i]], newdata=new.temp.df, se.fit=T)

preds.LLfit <- predict(ll_models[[i]], newdata=new.temp.df, se.fit=T)

preds.LAfit <- predict(la_models[[i]], newdata=new.temp.df, se.fit=T)

preds.LLAfit <- predict(lla_models[[i]], newdata=new.temp.df, se.fit=T)

preds.Mfit <- predict(m_models[[i]], newdata=new.temp.df.m, se.fit=T)

preds.MLfit <- predict(ml_models[[i]], newdata=new.temp.df.m, se.fit=T)

plot(Rangifer_tarandus ~ bio1, data=merged.global.dataset1, type="n", ylab="occurrence probability", cex.lab=1.75, 

     xlab="mean annual temperature [°C]", axes=F)

axis(1, cex.axis=1.25, labels=seq(-30, 30, by=1), at=seq(-30, 30, by=1))

axis(2, las=1, cex.axis=1.25)

box()

rug(merged.global.dataset1$bio1[merged.global.dataset1$Rangifer_tarandus==0], side=1, col=rgb(0.1,.1,.1,.1))

rug(merged.global.dataset1$bio1[merged.global.dataset1$Rangifer_tarandus==1], side=3, col=rgb(0.1,.1,.1,.1))

# Plot L predictions

polygon(c(new.temp.df$bio1, rev(new.temp.df$bio1)), plogis(c(preds.Lfit$fit+2*preds.Lfit$se.fit, 

                                                             rev(preds.Lfit$fit-2*preds.Lfit$se.fit))), col=rgb(.7,.7,.7,.15), border=NA)

lines(new.temp.df$bio1, plogis(preds.Lfit$fit), lwd=3, col="grey90")

# Plot LLA predictions

polygon(c(new.temp.df$bio1, rev(new.temp.df$bio1)), plogis(c(preds.LLAfit$fit+2*preds.LLAfit$se.fit, 

                                                             rev(preds.LLAfit$fit-2*preds.LLAfit$se.fit))), col=rgb(.5,.5,.5,.15), border=NA)

lines(new.temp.df$bio1, plogis(preds.Lfit$fit), lwd=3, col="grey70")

# plot M-fit:

polygon(c(new.temp.df.m$bio1, rev(new.temp.df.m$bio1)), plogis(c(preds.Mfit$fit+2*preds.Mfit$se.fit, 

                                                                 rev(preds.Mfit$fit-2*preds.Mfit$se.fit))), col=rgb(.1,.1,.1,.15), border=NA)

lines(new.temp.df.m$bio1, plogis(preds.Mfit$fit), lwd=3, col="grey30")

# plot ML-fit:

polygon(c(new.temp.df.m$bio1, rev(new.temp.df.m$bio1)), plogis(c(preds.MLfit$fit+2*preds.MLfit$se.fit, 

                                                                 rev(preds.MLfit$fit-2*preds.MLfit$se.fit))), col=rgb(.1,.1,.1,.15), border=NA)

lines(new.temp.df.m$bio1, plogis(preds.MLfit$fit), lwd=3, col="black")

legend("topright", legend="Reindeer", bty="n", cex=2)

legend("right", legend=c("L", "LLA", "M", "ML"), lwd=3, col=c("grey90", "grey70", "grey30", "black"), bty="n", cex=1.5)

## -- Precipitation -- ##

new.precip.df <- cbind.data.frame(bio12=seq(1, 4000, len=2000), t(apply(merged.global.dataset1[,c(4:5,7)], 2, median,na.rm=T)))

new.precip.df.m <- cbind.data.frame(bio12=seq(1, 4000, len=2000), t(apply(merged.global.dataset100[,c(4:5,7)], 2, median,na.rm=T)))

i<-11

preds.Lfit <- predict(l_models[[i]], newdata=new.precip.df, se.fit=T)

preds.LLfit <- predict(ll_models[[i]], newdata=new.precip.df, se.fit=T)

preds.LAfit <- predict(la_models[[i]], newdata=new.precip.df, se.fit=T)

preds.LLAfit <- predict(lla_models[[i]], newdata=new.precip.df, se.fit=T)

preds.Mfit <- predict(m_models[[i]], newdata=new.precip.df.m, se.fit=T)

preds.MLfit <- predict(ml_models[[i]], newdata=new.precip.df.m, se.fit=T)

backtransfBio12 <- (new.precip.df$bio12^2)-1

quantile(untransformedData1$bio12, 0.99, na.rm=T)

# use untransformed dataset for presence absence plotting

plot(get(names(l_models)[i]) ~ bio12, data=untransformedData1, type="n", xlim=(c(0,1631)), ylab="occurrence probability", cex.lab=1.75, 

     xlab="annual precipitation [mm]", axes=F)

axis(1, cex.axis=1.25, labels=seq(1, 1631, by=100), at=seq(1, 1631, by=100))

axis(2, las=1, cex.axis=1.25)

box()

rug(untransformedData1$bio12[untransformedData1$Rangifer_tarandus==0], side=1, col=rgb(0.1,.1,.1,.1))

rug(untransformedData1$bio12[untransformedData1$Rangifer_tarandus==1], side=3, col=rgb(0.1,.1,.1,.1))

# Plot L predictions

polygon(c(backtransfBio12, rev(backtransfBio12)), plogis(c(preds.Lfit$fit+2*preds.Lfit$se.fit, 

                                                           rev(preds.Lfit$fit-2*preds.Lfit$se.fit))), col=rgb(.7,.7,.7,.15), border=NA)

lines(backtransfBio12, plogis(preds.Lfit$fit), lwd=3, col="grey90")

# Plot LLA predictions

polygon(c(backtransfBio12, rev(backtransfBio12)), plogis(c(preds.LLAfit$fit+2*preds.LLAfit$se.fit, 

                                                           rev(preds.LLAfit$fit-2*preds.LLAfit$se.fit))), col=rgb(.5,.5,.5,.15), border=NA)

lines(backtransfBio12, plogis(preds.Lfit$fit), lwd=3, col="grey70")

# plot M-fit:

backtransfBio12m <- (new.precip.df.m$bio12^2)-1

polygon(c(backtransfBio12, rev(backtransfBio12)), plogis(c(preds.Mfit$fit+2*preds.Mfit$se.fit, 

                                                           rev(preds.Mfit$fit-2*preds.Mfit$se.fit))), col=rgb(.1,.1,.1,.15), border=NA)

lines(backtransfBio12, plogis(preds.Mfit$fit), lwd=3, col="grey30")

# plot ML-fit:

polygon(c(backtransfBio12, rev(backtransfBio12)), plogis(c(preds.MLfit$fit+2*preds.MLfit$se.fit, 

                                                           rev(preds.MLfit$fit-2*preds.MLfit$se.fit))), col=rgb(.1,.1,.1,.15), border=NA)

lines(backtransfBio12, plogis(preds.MLfit$fit), lwd=3, col="black")

legend("topright", legend="Reindeer", bty="n", cex=2)

legend(0,1.02, legend=c("L", "LLA", "M", "ML"), lwd=3, col=c("grey90", "grey70", "grey30", "black"), bty="n", cex=1.5)

## -- Predicted and original range size – ##

# Datasets are are already loaded from previous point

predRangeSize <- as.data.frame(matrix(nrow=12,ncol=9))

colnames(predRangeSize) <- c("species", "L_originalSize", "M_originalSize", "l","la","ll","lla","m","ml")

predRangeSize$species <- names(l_models)

# Calculate the original and predicted range size

for (i in 1:12){

  predRangeSize[i, "L_originalSize"] <- sum(merged.global.dataset1[,names(l_models)[i]] * merged.global.dataset1$cell_area, na.rm=T)

  predRangeSize[i, "M_originalSize"] <- sum(merged.global.dataset100[,names(l_models)[i]] * 10000, na.rm=T)

  predRangeSize[i, "l"] <- sum(plogis(predict(l_models[[i]], newdata=merged.global.dataset1)) * merged.global.dataset1$cell_area, na.rm=T)

  predRangeSize[i, "ll"] <- sum(plogis(predict(ll_models[[i]], newdata=merged.global.dataset1)) * merged.global.dataset1$cell_area, na.rm=T)

  predRangeSize[i, "la"] <- sum(plogis(predict(la_models[[i]], newdata=merged.global.dataset1)) * merged.global.dataset1$cell_area, na.rm=T)

  predRangeSize[i, "lla"] <- sum(plogis(predict(lla_models[[i]], newdata=merged.global.dataset1)) * merged.global.dataset1$cell_area, na.rm=T)

  predRangeSize[i, "m"] <- sum(plogis(predict(m_models[[i]], newdata=merged.global.dataset100)) * 10000, na.rm=T)

  predRangeSize[i, "ml"] <- sum(plogis(predict(ml_models[[i]], newdata=merged.global.dataset100)) * 10000, na.rm=T)

}

predRangeSize[,2:9] <- predRangeSize[,2:9]/1000000 (expressed as 10^6km)

predRangeSize

boxplot(predRangeSize[2:9],ylab="range size")

#########################

##### Relative bias #####

#########################

# Bias for every parameter estimate, for every species

# Rel bias species A = ||coeff_i_model_i| - |coeff_i_model_ref|| / se(coef_i_model_ref)|

# The smaller the standard error of the reference model, the higher the bias.

# The second formula weights each parameter by it's partial explained deviance in the model

# Important parameters will have a greater contribution to bias estimate.

# Function to calculate relative bias

calcBias <- function(x,y){

  # Relative bias for every parameter of model x, for species i

  paramRelBias <- abs((x$coefficients[-1] - y$coefficients[-1]) / coef(summary(y))[-1,2] )

  # now every parameter in the model x has to be weighted by its deviance (e.g. temp * (deviance temp)/(sum of deviances of all parameters))

  # parameters of less importance will have lower weight in the model

  # the mean bias of all parameters is then calculated for each species

  result <- mean(paramRelBias * anova(x)[-1,2]/sum(anova(x)[-1,2]))

  return(result)

}

relBias <- as.data.frame(matrix(nrow=12,ncol=5),row.names=names(l_models))

colnames(relBias) <- c("l","la","ll","lla","m")

models <- c("l","la","ll","lla","m")

for(i in models){

  relBias[,i] <- mapply(calcBias, get(paste(i,"_models",sep="")), ml_models) 

}

summary(relBias)

# -- Extracting parameters for relative bias analyses -- ## 

# min, max, mean, median latitude and latitude range of each species

range_lat <- as.data.frame(matrix(nrow=12, ncol=5), row.names=names(merged.global.dataset1)[10:21])

colnames(range_lat) <- c("minLat","maxLat","meanLat","medianLat", "rangeSize")

for(i in 1:12){

  range_lat[i,] <- round(c(min(merged.global.dataset1$y[merged.global.dataset1[rownames(range_lat)[i]]==1],na.rm=T), 

                           max(merged.global.dataset1$y[merged.global.dataset1[rownames(range_lat)[i]]==1],na.rm=T),

                           mean(merged.global.dataset1$y[merged.global.dataset1[rownames(range_lat)[i]]==1],na.rm=T),

                           median(merged.global.dataset1$y[merged.global.dataset1[rownames(range_lat)[i]]==1],na.rm=T),

                           sum(merged.global.dataset1[which(merged.global.dataset1[,rownames(range_lat)[i]]==1), "cell_area"])))

}

## -- Find the R-sq for each model -- ##

load("GLM_models.Rdata")

Rsq <- as.data.frame(matrix(nrow=12, ncol=5), row.names=names(l_models))

colnames(Rsq) <- c("l","la","ll","lla","m")

for(i in colnames(Rsq)){

  Rsq[,i] <- sapply(get(paste(i,"_models",sep="")), function(x) 1-(x$deviance/x$null.deviance)) 

}

## -- Stacking -- ## 

Rsq_stack <- data.frame(stack(Rsq), species=rownames(Rsq))

colnames(Rsq_stack)[1] <- "Rsq"

Rsq_stack$ID <- paste(Rsq_stack$species,Rsq_stack$ind, sep="") # Create and ID

Rsq_stack<- Rsq_stack[,c("ID","Rsq")]

relB <- data.frame(stack(relBias), species = rownames(relBias))

colnames(relB)[1:2] <- c("relBias", "model")

relB$proj <- c(rep("long-lat",48),rep("moll",12)) # add projections

relB$ID <- paste(relB$species, relB$model, sep="") # Create an ID for merging without errors

# Merge them

relBias <- merge(relB, Rsq_stack, by= "ID")

# Add other variables

range_lat$species <- rownames(range_lat)

biasAnalysis <- merge(relBias, range_lat, by="species")

biasAnalysis <- biasAnalysis[order(biasAnalysis$model),]

# Manually add other variables

biasAnalysis$LandcoverCorr <- c(rep(0,24),rep(1,24),rep(0,12))

biasAnalysis$AreaCorr <- c(rep(0,12),rep(1,12),rep(0,12),rep(1,24))

biasAnalysis <- biasAnalysis[order(biasAnalysis$species),]

biasAnalysis$coastal <- c(rep(1,5),rep(0,5),rep(1,10),rep(0,5),rep(1,10),rep(0,5),rep(1,10),rep(0,5),rep(1,5))

biasAnalysis$rangeLat <- biasAnalysis$maxLat - biasAnalysis$minLat

###############################

## ----- Bias analysis ----- ##

###############################

## -- bias among models -- ##

fm <- lm(log(relBias+0.01) ~ model, data=biasAnalysis)

anova(fm)

TukeyHSD(aov(fm)) 

## -- Mixed effect models -- ##

require(effects)

require(nlme)

#manually simplified:

fm <- lme(log(relBias+0.01) ~ maxLat +log(rangeSize) +LandcoverCorr * coastal   + proj + AreaCorr, random=~1|species, data=biasAnalysis)  

anova(fm)

summary(fm)

boxplot(relBias ~ model,data=biasAnalysis, whisklty=1, col="grey50", las=1)

# Find the mean relative bias for each projection

aggregate(.~model, FUN=mean, data=biasAnalysis[, 3:4])

aggregate(.~species, FUN=mean, data=biasAnalysis[, c(1,3)]) # elk and reindeer

## -- figure: individual biases ~ distribution size -- ##

attach(biasAnalysis)

rangeSize <- rangeSize/1000000

col <- sapply(model, function(x) switch(x, "l"="white", "ll"="grey90", "la"="grey50", "lla"="grey20", "m"="grey70"))

sym <- sapply(model, function(x) switch(x, "l"=21, "ll"=21, "la"=21, "lla"=21, "m"=22))

par(mar=c(5,5,1,1))

plot(biasAnalysis$relBias ~ rangeSize, las=1, bg=col, pch=sym, cex=2, log="x", type="n", xlab=expression(paste("range size [", 10^6, " ", km^2, "]")), 

     ylab="relative bias", cex.lab=1.6, cex.axis=1.25,mgp=c(3.5,0.5,0))

abline(h=0, lwd=2, col="grey")

points(biasAnalysis$relBias ~ rangeSize, bg=col, pch=sym, cex=2)

legend("topleft", bty="n", pch=c(21, 21, 21, 21, 22), pt.bg=c("white", "grey90", "grey50", "grey20", "grey70"), legend=c("L", "LL", "LA", "LLA", "M"), cex=1.5)

text(tapply(rangeSize, species, max), tapply(biasAnalysis$relBias, species, max)+0.01+c(0,0,-0.12,0,0,0,0,-0.1,0,0,0,-0.08),

     c("Eurasian elk", "Bactrian camel *",  "Siberian roe deer", "Red deer", 

       "Alpine musk deer *", "Mule deer", "Muskox", "Wild sheep *", "Bighorn sheep", "Thinhorn sheep", "Mongolian gazelle *","Reindeer"), 

     srt=90, pos=4, offset=-.1, font=3, cex=0.9)

## -- figure: bias for each model -- ##

biasAnalysis$model <- factor(biasAnalysis$model, levels=c("l", "ll", "la", "lla", "m"))

plot(biasAnalysis$model,biasAnalysis$relBias,at=c(1,2,3,4,5),type="n", ylab="relative bias [SD multiples]", cex.lab=2,whisklty=1,col=c("white", "grey90", "grey70", "grey50", "grey45"),axes=F,xlab="model variants", cex.lab=1.4)

abline(h=0, col="grey", lwd=2)

axis(side=2, las=1)

axis(1,at=1:5, labels=c("L", "LL", "LA", "LLA", "M"),tick=F)

box()

## -- Additional analyses: model differences – ##

## -- Coefficients SE among models -- ##

# Relative bias calculation is influenced by the coeffient standard error of in the reference model (ML).

# We test here whether standard errors differ among coefficients in different model variants.

errorEst <- as.data.frame(matrix(nrow=72,ncol=10))

colnames(errorEst) <- c("model", "species","bio1","bio1sq","bio2","bio2sq","bio12","bio12sq","bio15","bio15sq")

errorEst$species <- rep(names(l_models), 6)

errorEst$model <- rep(c("l","la","ll","lla","m","ml"),each=12)

errorEst$proj <- c(rep("variant",60),rep("ref",12))

for(i in unique(errorEst$model)){

  errorEst[which(errorEst$model==i),3:10] <-t(sapply(get(paste(i,"_models",sep="")), function(x) coef(summary(x))[-1,2]))

}

# linear model coefficientSE ~ model_variant

apply(errorEst[,3:10], 2, function(x) anova(lm(x~model,data = errorEst)))

## -- Predictors partial explained deviances -- ##

paramWeights <- as.data.frame(matrix(nrow=72,ncol=11))

colnames(paramWeights) <- c("model","areaWeight", "species","bio1","bio1sq","bio2","bio2sq","bio12","bio12sq","bio15","bio15sq")

paramWeights$species <- rep(names(l_models), 6)

paramWeights$model <- rep(c("l","la","ll","lla","m","ml"),each=12)

paramWeights$areaWeight <- c(rep(0,12),rep(1,12),rep(0,12),rep(1,36))

paramWeights$proj <- c(rep("l",48),rep("m",24))

for(i in unique(paramWeights$model)){

  paramWeights[which(paramWeights$model==i),4:11] <-t(sapply(get(paste(i,"_models",sep="")), function(x) anova(x)[-1,2]/sum(anova(x)[-1,2])))

}

attach(paramWeights)

library(nlme)

anova(flm1 <- lme(bio1 + bio1sq ~ areaWeight, random=~1|species, data=paramWeights))

anova(lme(bio2 +bio2sq ~ areaWeight, random=~1|species, data=paramWeights)) 

anova(lme(bio12 +bio12sq~ areaWeight, random=~1|species, data=paramWeights))

anova(lme(bio15+bio15sq ~ areaWeight, random=~1|species, data=paramWeights))

# bio2, bio12 and bio15 at 0.05 level differ between with/our areaWeight,

# bio2 differ among projection types (M/L) (bio12 and bio15 marginally 0.06-0.07)

# bio2 differ among models

## -- Predictors absolute estimates among models -- ##

paramEst <- as.data.frame(matrix(nrow=72,ncol=10))

colnames(paramEst) <- c("model", "species","bio1","bio1sq","bio2","bio2sq","bio12","bio12sq","bio15","bio15sq")

paramEst$species <- rep(names(l_models), 6)

paramEst$model <- rep(c("l","la","ll","lla","m","ml"),each=12)

paramEst$proj <- c(rep("l",48),rep("m",24))

for(i in unique(paramEst$model)){

  paramEst[which(paramEst$model==i),3:10] <-t(sapply(get(paste(i,"_models",sep="")), function(x) abs(x$coefficients[-1])))

}

summary(flm1 <- lme(bio1 ~ proj, random=~1|species, data=paramEst))

summary(lme(bio1sq ~ proj, random=~1|species, data=paramEst))

summary(lme(bio2 ~ proj, random=~1|species, data=paramEst))

summary(lme(bio2sq ~ proj, random=~1|species, data=paramEst))

summary(lme(bio12 ~ proj, random=~1|species, data=paramEst))

summary(lme(bio12sq ~ proj, random=~1|species, data=paramEst))

summary(lme(bio15 ~ proj, random=~1|species, data=paramEst))

summary(lme(bio15sq ~ proj, random=~1|species, data=paramEst))

# so bio1 and bio1sq  differ between projections, bio15 at 0.05 level

### additional test: if regression dillution occurrs more in non-area-weighted models among lon-lat versions -- NO, it does not

predict(flm1, newdata=data.frame("proj"=c("l","m")), level=0) # difference between with/out area-correction

library(AICcmodavg)

preds <- predictSE.lme(flm1, newdata=data.frame("proj"=c("1","m"))) # population level

barplot(preds$fit, ylim=c(0, .5), las=1, names.arg = c("l", "m"), ylab="parameter estimate for bio1", cex.lab=2)

arrows(0.7, preds$fit[1]-preds$se.fit[1], 0.7, preds$fit[1]+preds$se.fit[1], code=3, angle=90, lwd=2)

arrows(1.9, preds$fit[2]-preds$se.fit[2], 1.9, preds$fit[2]+preds$se.fit[2], code=3, angle=90, lwd=2)
